# RNA splicing factors and long noncoding RNA reciprocally regulate EGFRvIII expression in human cancer cells

**DOI:** 10.1186/s12929-026-01298-z

**Published:** 2026-11-11

**Authors:** Hsuan-Yu Peng, Jang-Yang Chang

**Affiliations:** TMU Research Center of Cancer Translational Medicine, Taipei Medical University Hospital, College of Medicine, Taipei Medical University, No. 252, Wuxing St., Xinyi Dist., Taipei, 110301 Taiwan (R.O.C.)

**Keywords:** EGFRvIII, Alternative splicing, Long noncoding RNA, *RP11-30P6.6*, SRSF1, microRNA, Oral squamous cell carcinoma, Carcinogen exposure

## Abstract

**Background:**

Dysregulated epidermal growth factor receptor variant III (EGFRvIII) contributes to tumor progression in several human cancers, including oral squamous cell carcinoma (OSCC) and glioblastoma multiforme. Although genomic alterations are frequently associated with EGFRvIII expression, the contribution of environmental carcinogens and RNA-based regulatory mechanisms to its expression remains poorly understood and was investigated in this study.

**Methods:**

Chronic exposure models of the tobacco-specific nitrosamine nicotine-derived nitrosamine ketone (NNK) and the areca nut alkaloid arecoline were established in dysplastic oral keratinocytes. Expression and splicing patterns of EGFR isoforms were analyzed using molecular assays and long-read sequencing. Functional assays, RNA pull-down, AGO2 immunoprecipitation, MS2-based RNA affinity purification, and reporter assays were performed to investigate the roles of splicing factors, long noncoding RNAs (lncRNAs), and microRNAs in EGFRvIII regulation.

**Results:**

Carcinogen exposure induced sustained EGFR and EGFRvIII expression, increased the levels of exon 1–8 junction-containing transcripts, and expanded EGFR isoform diversity. These changes were accompanied by upregulation of splicing regulators, particularly SRSF1. Transcriptomic and functional analyses identified the lncRNA *RP11-30P6.6* as a carcinogen-responsive factor that promoted cell proliferation and migration and sustained increased EGFRvIII expression. RNA affinity-based assays showed preferential recovery of SRSF1 and EGFRvIII-related RNA in *RP11-30P6.6* pull-down fractions, consistent with its involvement in EGFRvIII-associated RNA regulation. Functional and AGO2 immunoprecipitation analyses were consistent with a competing endogenous RNA (ceRNA)-like relationship in which *RP11-30P6.6* may attenuate repression by candidate microRNAs that showed differential regulatory effects on EGFRvIII relative to WT-EGFR under the conditions tested. Perturbation of SRSF1, *RP11-30P6.6*, or candidate microRNAs attenuated EGFRvIII expression, while functional modulation of these regulatory components was associated with altered tumor cell growth and migration.

**Conclusion:**

The study findings support a model in which environmental carcinogens reprogram RNA splicing and noncoding RNA networks to sustain EGFRvIII expression. SRSF1, *RP11-30P6.6*, and microRNAs may contribute to the post-transcriptional regulation of EGFRvIII expression and may provide potential targets for EGFRvIII-associated cancers.

**Graphical Abstract:**

**Figure:**
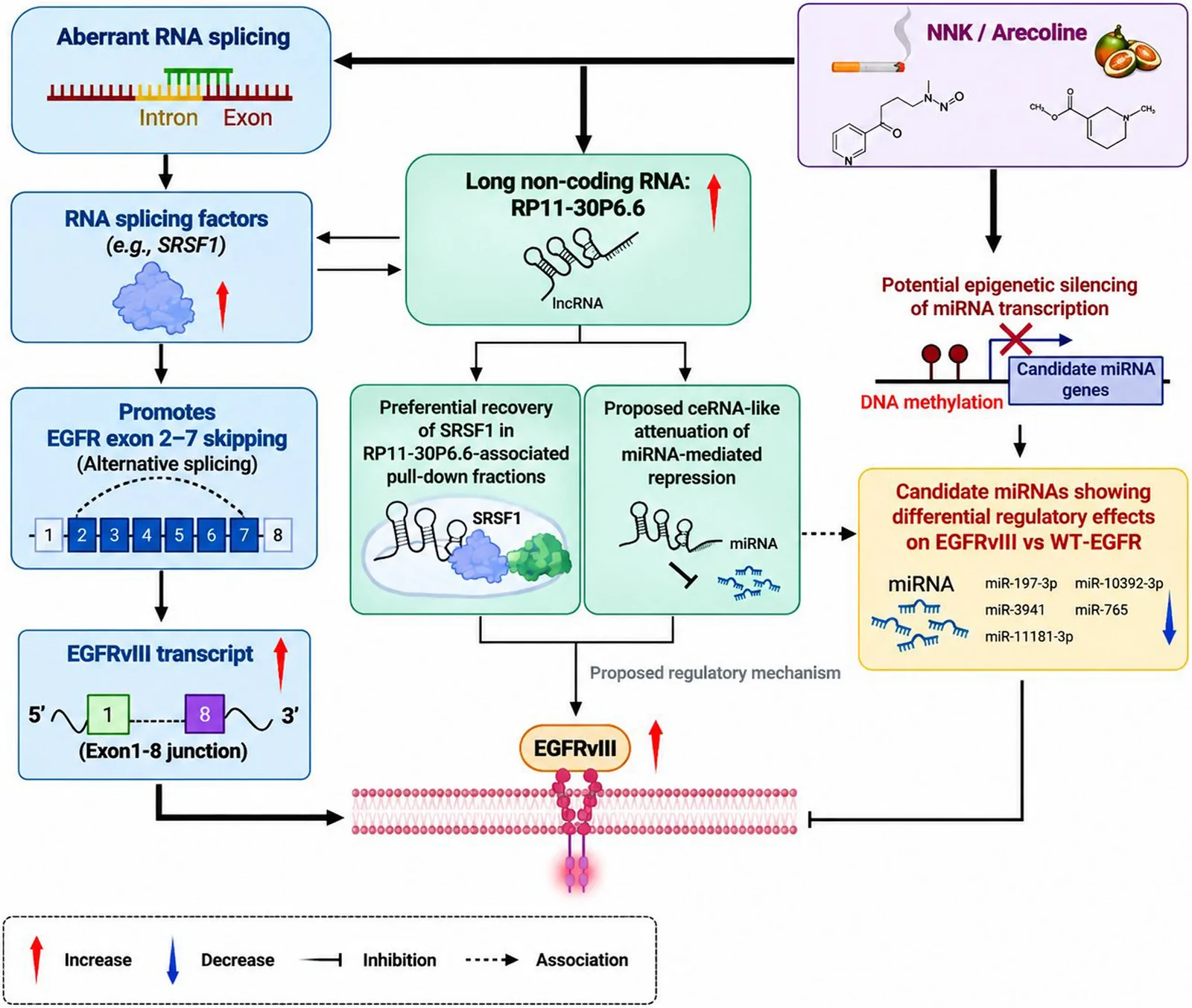

**Supplementary Information:**

The online version contains supplementary material available at https://doi.org/10.1186/s12929-026-01298-z.

## Background

Epidermal growth factor receptor (EGFR) is a central oncogenic driver in epithelial malignancies, including non-small cell lung cancer (NSCLC), glioblastoma multiforme (GBM), and head and neck squamous cell carcinoma (HNSCC) [45, 48, 53]. Beyond kinase-activating mutations and amplifications, EGFR pre-mRNA can undergo alternative splicing to generate soluble or truncated isoforms [65, 66]. In GBM and some epithelial cancers, intragenic rearrangements involving deletion of exons 2–7 generate the truncated variant EGFRvIII, which retains constitutive kinase activity and has been associated with increased proliferation, invasion, and resistance [10, 34, 58]. In HNSCC, genomic deletion, rather than pure exon skipping, appears to underlie most EGFRvIII transcripts [58], but splicing factors and RNA-binding proteins (RBPs) may influence EGFRvIII transcript processing, export, and stability of EGFRvIII transcripts generated from such genomic lesions [11, 42, 44].

Oral squamous cell carcinoma (OSCC) is a major HNSCC subtype, in which EGFR overexpression or hyperactivation commonly correlates with aggressive clinical behavior and poor response to therapy [45, 52]. Key epidemiological drivers of OSCC include chronic tobacco use, betel quid/areca nut chewing, alcohol consumption, and high-risk human papillomavirus infection [22]. Areca nuts deliver carcinogenic alkaloids, such as arecoline and nitrosamines formed during chewing, whereas tobacco smoke provides tobacco-specific nitrosamines, such as nicotine-derived nitrosamine ketone (NNK) [12, 56, 61]. These agents induce DNA damage and epigenetic remodeling, with accumulating evidence linking them to deregulated RNA splicing and non-coding RNA (ncRNA) expression [18, 27, 65, 66]. Therefore, OSCC provides a clinically relevant setting in which environmental carcinogens converge on EGFR-driven signaling and RNA-processing pathways with the potential to influence EGFR isoform usage.

MicroRNAs (miRNAs) and long noncoding RNAs (lncRNAs) are also increasingly being recognized as regulators of EGFR signaling [7, 13]. NcRNAs modulate the EGFR axis by targeting *EGFR* mRNA, ERBB family members, or downstream nodes, thereby influencing tumor initiation, progression, and resistance to EGFR tyrosine kinase inhibitors (TKIs) [6, 26, 54]. In addition to their effects on receptor and pathway components, ncRNAs can modulate splicing by scaffolding or titrating splicing factors, altering chromatin at splice-relevant loci, and regulating the abundance of splicing factors through competing endogenous RNA (ceRNA) mechanisms [9, 33, 46]. LncRNAs in HNSCC can remodel chromatin, transcription, and mRNA processing to affect EGFR signaling [5, 21]. Together, these findings suggest that ncRNAs may provide a regulatory link between EGFR signaling and the splicing machinery.

The multilayered regulatory networks described above are particularly relevant to carcinogen-associated OSCC, in which tobacco-derived nitrosamines and areca-related alkaloids remodel gene expression, miRNA profiles, and alternative splicing programs [19, 60, 61]. However, how carcinogen exposure integrates EGFR genomic alterations, alternative splicing, and ncRNA networks to control the abundance and signaling output of EGFRvIII in OSCC remains unclear [59]. Existing data support a framework in which the presence of *EGFRvIII* is not an isolated genomic event, but provides a node within a triangular network of intragenic rearrangements, splicing-factor activity, and ncRNA regulation. Intragenic deletions or *EGFR::EGFR* fusions generate an exon 1–8 junction that gives rise to the *EGFRvIII* pre-mRNA. Its processing and translation are influenced by splicing and RNA-processing factors, which may, in turn, remodel splicing-factor and ncRNA expression through transcriptional regulators, such as STAT3 and NF-κB [5, 43, 62, 64]. We hypothesized that carcinogen-induced reprogramming of splicing factors and ncRNA networks contributes to the establishment of an EGFRvIII-associated regulatory network in OSCC. In the present study, we investigated a regulatory network involving the splicing regulator SRSF1, the lncRNA *RP11-30P6.6*, and associated miRNAs, and evaluated their contributions to *EGFRvIII* regulation.

## Materials and methods

### Cell lines

Human dysplastic oral keratinocyte (DOK) cells (RRID: CVCL_1180), human glioblastoma cell lines U87 and A172 (a gift from Dr. Tsung-I Hsu, International Master Program in Medical Neuroscience, College of Medical Science and Technology, Taipei Medical University), and human OSCC cell line CAL 27 (Cat# CRL-2095; RRID: CVCL_1107) were obtained from the American Type Culture Collection (ATCC, Rockville, MD, USA) and cultured in Dulbecco’s modified Eagle’s medium (DMEM; Gibco, Grand Island, NY, USA). Carcinogen-transformed DOK derivative lines were established and maintained as previously described [4] in the laboratory of Dr. Ching-Chuan Kuo (Institute of Biotechnology and Pharmaceutical Research, National Health Research Institutes, Taiwan). Briefly, parental DOK cells were continuously exposed to noncytotoxic concentrations of NNK (10 μM) or arecoline (50 μM for 12 months, and the surviving transformed cells were selected, expanded, and designated as NNK-tolerant (NNKL and arecoline-tolerant (AL cell lines. OSCC cell lines HSC-2 (Cat# JCRB0622; RRID: CVCL_1287, HSC-3 (Cat# JCRB0623; RRID: CVCL_1288, the highly metastatic derivative HSC-3-M3 (Cat# JCRB1354; RRID: CVCL_8323, and HSC-4 (Cat# JCRB0624; RRID: CVCL_1289 were obtained from the Japanese Collection of Research Bioresources (JCRB, Tokyo, Japan and were maintained in α-minimum essential medium (α-MEM; Gibco). The human OSCC cell line SAS (Cat# JCRB0620; RRID: CVCL_1675) was obtained from JCRB and cultured in high-glucose DMEM. Human tongue/oral squamous carcinoma cell lines SCC-4 (Cat# CRL-1624; RRID: CVCL_1684), SCC-9 (Cat# CRL-1629; RRID: CVCL_1685), SCC-15 (Cat# CRL-1623; RRID: CVCL_1681), and SCC-25 (Cat# CRL-1628; RRID: CVCL_1686) were obtained from ATCC and maintained in a 1:1 mixture of DMEM and Ham’s F-12 (DMEM/F-12), supplemented with 1 mM sodium pyruvate and 400 nM hydrocortisone. Human embryonic kidney 293 T cells (HEK293T; RRID: CVCL_0063) were obtained from ATCC and cultured in DMEM. Unless otherwise specified, all media were supplemented with 10% fetal bovine serum (FBS), 1% penicillin–streptomycin (PS), and 1% non-essential amino acids (NEAA) (Gibco). Cells were maintained at 37 °C in a humidified atmosphere containing 5% CO_2_ and were used within 3 months of resuscitation from frozen stocks, with fewer than 20 passages per experiment. Cell line authentication was performed within the past three years via morphology and/or short tandem repeat (STR) profiling, and the resulting DNA profiles were compared with reference STR databases to confirm identity. All cultures were routinely tested for mycoplasma contamination and monitored microscopically for morphological integrity.

### Analysis of *EGFRvIII* isoform expression

Total RNA was isolated from the cultured cell lines using TRIzol reagent (Life Technologies, Gaithersburg, MD, USA) according to the manufacturer’s instructions. The RNA concentration and purity were determined by measuring the A260/280 ratio using a NanoDrop spectrophotometer (BRED-1000, Blue-Ray). *EGFR* transcripts are frequently altered through splicing or deletion events, with variant III (EGFRvIII), characterized by an in-frame deletion of exons 2–7, being the most common oncogenic isoform. To detect *EGFRvIII* expression, 100 ng of total RNA was reverse-transcribed using the RevertAid RT Reverse Transcription Kit (Cat# K1691, Thermo Scientific, USA) following the manufacturer’s protocol. PCR amplification of *EGFR* exons 1 to 8 was performed using the following primers: forward 5′-GGGCTCTGGAGGAAAAGAAA-3′ and reverse 5′-AGGCCCTTCGCACTTCTTAC-3ʹ. The cycling conditions were as follows: initial denaturation at 95 °C for 2 min, followed by 30 cycles of denaturation at 95 °C for 30 s, annealing at 55 °C for 45 s, and extension at 72 °C for 90 s, and a final extension at 72 °C for 5 min. RT-PCR products were resolved on 0.8% agarose gels; a 128 bp band indicated the presence of *EGFRvIII*. Primer sequences and amplification strategies were adapted from previously described methods [20].

For specific detection of minigene-derived exon 1–exon 8 junction transcripts, a separate minigene-specific primer pair was used. The forward primer spanning the exon 1–exon 8 junction was 5′-CGGGCTCTGGAGGAAAAGAAAGGTAATT-3′, and the reverse primer spanning the exon 8–FLAG junction of the minigene construct was 5′-CTTTGTAGTCCTTTGCGGCAAGGC-3′. This primer design enabled specific detection of the minigene-derived exon 1–exon 8 junction transcript and distinguished it from endogenous *EGFR* transcripts. The same minigene-specific primer pair was used for RT-PCR and qRT-PCR analyses. The identity of the minigene-derived exon 1–exon 8 junction product was confirmed by Sanger sequencing.

### Plasmids, esiRNA, and transfection

For *RP11-30P6.6* knockdown, DOK-AL, DOK-NNKL, HSC-2, and HSC-3 cells were transfected with a MISSION^®^ long noncoding esiRNA targeting human *RP11-30P6.6* (Ensembl Gene ID: ENSG00000234155; GenBank accession no. AL135903; Cat. No. EHNC004251; Sigma-Aldrich, Merck, NJ, USA) using TransIT-X2 Transfection Reagent (Mirus Bio, Madison, WI, USA) according to the manufacturer’s instructions. The MISSION^®^ esiRNA consists of a heterogeneous pool of endoribonuclease-prepared siRNAs targeting a defined region of the *RP11-30P6.6* transcript. DOK-AL, DOK-NNKL, and OSCC cells (HSC-3-M3) were transfected with ON-TARGETplus siRNA SMARTpools targeting splicing regulators (SRSF1, SRSF7, PTBP1, DDX21, and DDX60; Horizon Discovery, Cambridge, UK), including siSRSF1 (L-018672-01), siSRSF7 (L-015909-00), siPTBP1 (L-003528-00), siDDX21 (L-011919-01), siDDX60 (L-017664-01), or a nontargeting control siRNA (D-001810-10-05) using TransIT-X2 under identical conditions. Unless otherwise indicated, siRNAs were used at a final concentration of 50 nM and the cells were harvested 48–72 h after transfection for RNA and protein analyses. For lentiviral production, 293 T cells were cotransfected with pMD.G, pCMVΔR8.91, and lentiviral transfer plasmids using TransIT-LT1 (Mirus Bio). Viral supernatants were collected 48 h after transfection, clarified via centrifugation at 500 × *g* for 5 min, and filtered through a 0.45 μm filter. The supernatants were supplemented with polybrene (8 μg/mL) and used to infect target cells for 6 h.

### Luciferase reporter assay

Luciferase reporter assays were performed 48 h after transfection with an empty control vector, pmiGLO constructs containing partial *EGFR* or *EGFRvIII* coding sequences, or *EGFR* minigene reporters cloned into pGL3. Firefly and Renilla luciferase activities were measured using the Dual-Luciferase Reporter Assay System (Promega, Madison, WI, USA), according to the manufacturer’s instructions, on a GloMax Navigator microplate luminometer (Promega). Firefly luciferase activity was normalized to that of Renilla luciferase to control for the transfection efficiency.

### AGO2 immunoprecipitation assay

To examine the association of *RP11-30P6.6* with AGO2-containing complexes, OSCC and 293 T cells were cotransduced with pHA-*His-AGO2* (a gift from Dr. Shine-Gwo Shiah, National Health Research Institutes, Taiwan) and pCMV6-*RP11-30P6.6* lentiviral particles for 48 h. The cells were washed with ice-cold phosphate-buffered saline (PBS) and lysed in lysis buffer containing 50 mM Tris–HCl (pH 7.4), 150 mM NaCl, 1% NP-40, 1 mM PMSF, and 1 × protease inhibitor cocktail for 10 min on ice. Lysates were clarified via centrifugation at 12,000 × *g* for 15 min at 4 °C, and protein concentration was determined using Bradford assay (Bio-Rad). For AGO2 pull-down, 2 mg of total protein was incubated with 50 μL nickel–NTA magnetic agarose beads (Qiagen) pre-equilibrated in wash buffer (20 mM sodium phosphate, 500 mM NaCl, 10 mM imidazole, pH 7.5) for 16 h at 4 °C with rotation to capture His-tagged AGO2 complexes. The beads were washed five times with cold lysis buffer and eluted with 250 mM imidazole. Eluates containing HA-His-AGO2 complexes were subjected to RNA extraction using the TRIzol LS reagent (Invitrogen, USA). RNA was precipitated with ethanol in the presence of glycogen (1 μL; Roche), washed with 70% ethanol, air-dried, and resuspended in diethyl pyrocarbonate-treated water. RNA concentration and purity were measured using an EzDrop 1000 UV/Vis spectrophotometer (Blue-ray Bio, Taiwan). RT-qPCR was performed to detect *RP11-30P6.6* and associated miRNAs (miR-197-3p, miR-3941, miR-11181-3p, miR-10392-3p, and miR-765).

### Immunofluorescence staining

Cells were fixed with freshly prepared 4% paraformaldehyde (Merck, Whitehouse Station, NJ, USA) for 10 min at room temperature, followed by permeabilization with 0.05% Triton X-100 in PBS for 5 min. The cells were blocked with 3% bovine serum albumin (BSA) in PBS at 37 °C for 1 h to minimize nonspecific binding. For SRSF1 staining, the cells were incubated overnight with a mouse anti-SRSF1 monoclonal antibody (sc-33652; Santa Cruz Biotechnology), diluted in 3% BSA/PBS, at 4 °C. The following day, the cells were washed with PBS and incubated with a Plus 594-conjugated secondary antibody (RGAM004, 1:500; Proteintech) for 2 h at room temperature in the dark. Finally, nuclei were counterstained with 4′, 6-diamidino-2-phenylindole (DAPI) for 5 min at room temperature. Coverslips were mounted using the ProLong Gold antifade reagent (Invitrogen, Carlsbad, CA, USA). Fluorescence images were captured using the EVOS M5000 Imaging System (Thermo Fisher Scientific, USA).

### RNA in situ hybridization

RNA in situ hybridization (RNA-FISH) was performed using the BioSpot ISH detection system (BioTnA) according to the manufacturer’s instructions, with minor modifications. The cells were cultured on glass coverslips and fixed with 4% paraformaldehyde in PBS for 10 min at room temperature, followed by permeabilization with 0.1% Triton X-100 in PBS for 5 min. For dual RNA detection, two probes were used simultaneously: a biotin-labeled probe targeting the wild-type *EGFR* (WT-*EGFR*) exon1–exon2 junction and a digoxigenin (DIG)-labeled probe pool targeting the lncRNA *RP11-30P6.6* transcript. The WT-*EGFR* probe specifically recognizes the exon1–exon2 junction of the canonical EGFR transcript, whereas the *RP11-30P6.6* probe pool targets multiple regions across the lncRNA transcript to enhance detection sensitivity. The probes were diluted to a final concentration of 10 μM in diethyl pyrocarbonate-treated water, mixed, and applied to the samples. After probe application, the slides were sealed with coverslips, and the samples were denatured at 96 °C for 10 min, followed by hybridization in a humidified chamber at 37 °C overnight. After hybridization, coverslips were removed by immersion in 1 × PBST wash buffer and slides were washed sequentially in 2 × and 1 × SSC buffers prewarmed to 45 °C for 10 min each to remove unbound probes. Signal detection was performed sequentially to minimize cross-reactivity. Biotin-labeled WT-*EGFR* probes were first detected using a BioSpot TAMRA detection kit, generating orange-red fluorescence signals. Slides were then re-blocked before detecting DIG-labeled *RP11-30P6.6* probes using an anti-DIG antibody followed by F488 labeling, producing green fluorescence signals. Nuclei were counterstained with DAPI for 1 min and the slides were mounted using an aqueous mounting medium. Fluorescence images were acquired using a widefield fluorescence microscope equipped with DAPI, FITC/FAM, and TRITC/TAMRA filter sets and a 20X objective lens. Images were captured sequentially in the order TAMRA → FAM → DAPI to minimize spectral overlap. Control experiments included single-probe hybridization and no-probe controls to verify the signal specificity and exclude channel crosstalk.

### RNA pull-down assay

RNA pull-down assays were performed to examine the preferential recovery of proteins and RNAs associated with biotin-labeled *RP11-30P6.6* under the pull-down conditions. Biotin-labeled *RP11-30P6.6* RNA and an unrelated control RNA were synthesized via T7-mediated in vitro transcription using the HiScribe T7 High Yield RNA Synthesis Kit (Cat. #E2040S, New England Biolabs, Ipswich, MA, USA) in the presence of the Biotin RNA Labeling Mix (Roche, Cat. #11685597910), according to the manufacturers’ instructions. The synthesized RNAs were purified using the MEGAclear Transcription Clean-Up Kit (Thermo Fisher Scientific), and RNA concentration and purity were determined using a NanoDrop spectrophotometer (BRED-1000, Blue-Ray).

For RNA pull-down assays, 2 μg of biotin-labeled RNA was incubated with 1 mg of total cell lysate from 293 T cells stably expressing EGFRvIII in lysis buffer (50 mM Tris–HCl, pH 7.5, 150 mM NaCl, 1 mM MgCl_2_, 0.5% NP-40, and 1 × protease inhibitor cocktail) for 2 h at 4 °C with gentle rotation. RNA–protein and RNA-associated complexes were captured using streptavidin magnetic beads (Thermo Fisher Scientific) for an additional 4 h at 4 °C. The beads were washed extensively with ice-cold lysis buffer, and the bound complexes were eluted by heating at 95 °C for 5 min in SDS sample buffer. For protein analysis, eluates were subjected to western blotting using the EGFRvIII-specific rabbit monoclonal antibody RM419 (Cat. No. 31–1305-00, RevMAb Biosciences), EGF Receptor (D38B1) Rabbit Monoclonal Antibody #4267 (Cell Signaling Technology), and an antibody against SRSF1 (A4091, ABclonal). For RNA analysis, RNA was extracted from the beads using TRIzol reagent (Thermo Fisher Scientific), followed by RT-qPCR to detect *EGFRvIII* and associated transcripts.

### GST-based MS2-TRAP pull-down

GST-based MS2-TRAP was used to isolate RNA–protein complexes assembled on MS2-tagged pre-mRNAs. 293 T cells were cotransfected with MS2-tagged *EGFR* minigenes (or other MS2-tagged RNA constructs) together with pcDNA3.1_NLS–FLAG–MCP–GGGGS–GST using TransIT-LT1 (Mirus Bio) according to the manufacturer’s instructions. A typical transfection used a plasmid mass ratio of 3:1 (MS2-tagged RNA construct: NLS–FLAG–MCP–GST), with a total of 10 μg of DNA per 10 cm dish. At 48 h after transfection, cells were washed with ice-cold PBS and lysed in 1 mL ice-cold lysis buffer [50 mM Tris–HCl (pH 7.4), 150 mM NaCl, 0.5% NP-40, 2 mM MgCl_2_, 1 mM DTT, 1 × protease inhibitor cocktail (Roche), and 1 U/μL RNase inhibitor (Promega]) for 10 min on ice. The lysates were clarified via centrifugation at 12,000 × *g* for 15 min at 4 °C, and 5% of the supernatant was used as the input. For MS2-TRAP purification, the cleared lysates were incubated with glutathione magnetic beads (G0924, Sigma-Aldrich) pre-equilibrated in lysis buffer for 2 h at 4 °C with rotation. Beads were washed 5–6 times with high-salt wash buffer (lysis buffer containing 300 mM NaCl and 0.05–0.1% NP-40), followed by a final wash with low-salt buffer (150 mM NaCl). For RNA analysis, the bead-bound complexes were treated with proteinase K (0.5 mg/mL in 1% SDS buffer) at 55 °C for 45 min. RNA was extracted using phenol–chloroform, precipitated with ethanol, treated with DNase I, reverse-transcribed, and analyzed via qRT-PCR to quantify MS2-tagged pre-mRNAs and associated noncoding RNAs. For protein analysis, GST pull-down samples were eluted by boiling the beads in 2 × Laemmli sample buffer containing 50 mM DTT, resolved via SDS–PAGE, and analyzed via immunoblotting for EGFR/EGFRvIII, splicing factors, and other proteins. The N-terminal NLS promotes nuclear accumulation of MCP and facilitates capture of nuclear MS2-tagged RNA-associated complexes.

### Statistical analysis

All experiments were performed with at least three independent biological replicates, as indicated in the figure legends. Data are presented as the mean ± standard deviation (SD). Differences between two groups were analyzed using unpaired two-tailed Student’s *t*-tests, and comparisons among multiple groups were assessed using one-way analysis of variance (ANOVA) with appropriate post-hoc tests. Statistical significance was set at *P* < 0.05. Statistical analyses were performed using GraphPad Prism version 8.0 (GraphPad Software, San Diego, CA, USA).

## Results

### NNK and arecoline induce EGFR and EGFRvIII expression in human cancer cell models

**Fig. 1:**
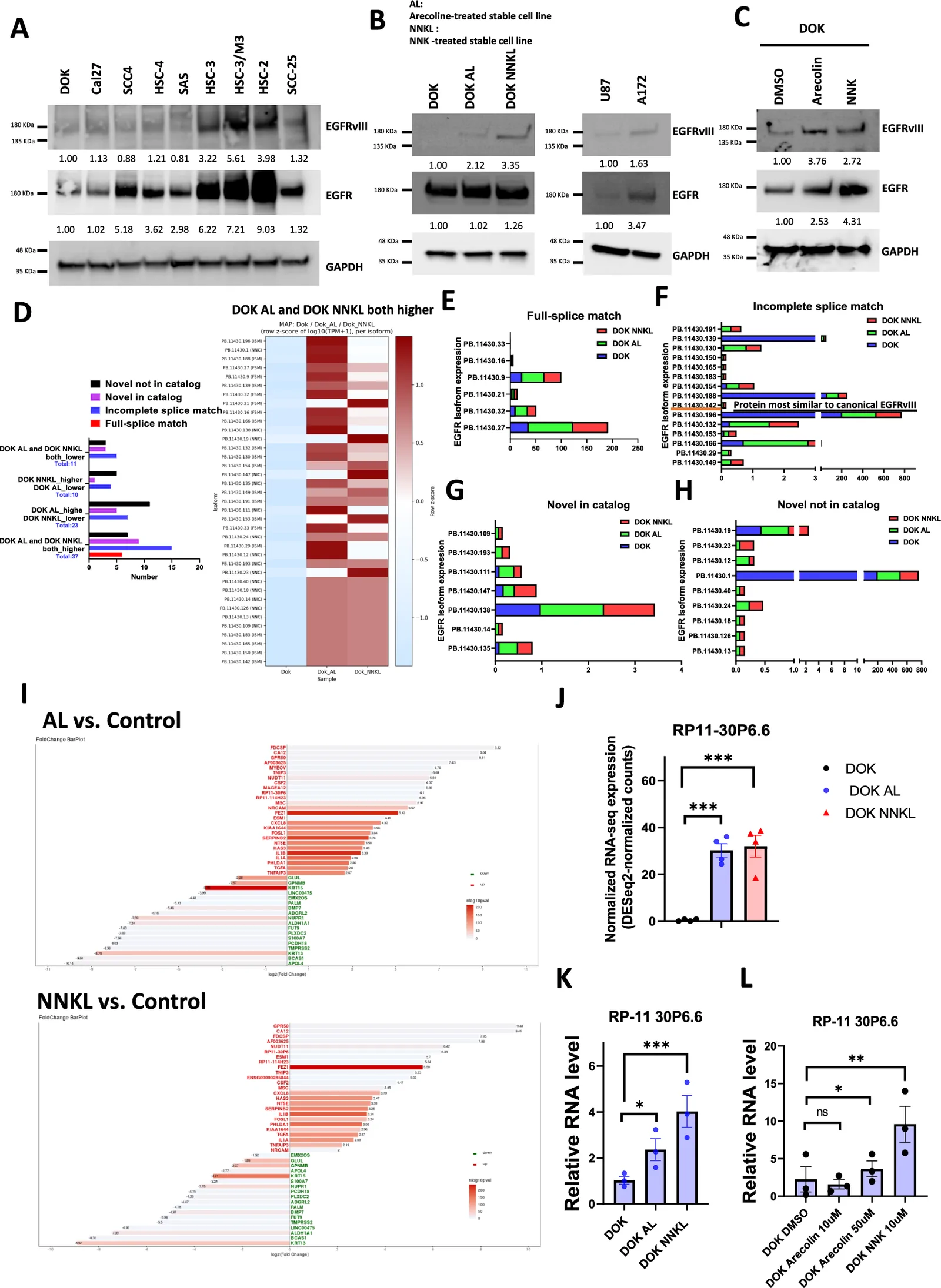
NNK and arecoline induce EGFRvIII expression and lncRNA upregulation in dysplastic oral keratinocytes. **A** Immunoblot analysis of EGFRvIII in OSCC cell lines. GAPDH was used as a loading control. Densitometric quantification, normalized to GAPDH, is shown beneath the blots. *n* = 2. **B** Immunoblot analysis of EGFR and EGFRvIII in DOK cells continuously exposed for 12 months to non-cytotoxic NNK (10 µM) or arecoline (50 µM), generating NNK-tolerant (NNK-L) and arecoline-tolerant (AL) derivatives, respectively, as well as in U87 and A172 cells. GAPDH was used as a loading control. *n* = 3. **C** Immunoblot analysis of EGFR and EGFRvIII in DOK cells treated with NNK (10 µM) or arecoline (50 µM) for 72 h. Data are representative of at least three independent experiments. GAPDH was used as a loading control. *n* = 3. **D**–**H** PacBio long-read amplicon sequencing of *EGFR* transcripts in parental DOK cells and carcinogen-adapted derivatives (DOK-AL and DOK-NNKL). The transcript categories shown in **D** represent classification based on relative abundance patterns across the three experimental groups and should not be interpreted as direct comparisons of total transcript abundance. The panel on the left (**D**) summarizes the number of transcript isoforms classified according to their relative abundance patterns across DOK, DOK-AL, and DOK-NNKL cells. The heatmap (right panel) displays row-wise Z-score-normalized log_10_(TPM + 1) values for individual transcript isoforms and illustrates relative expression patterns rather than absolute transcript abundance. *EGFR* transcript isoforms were classified as **E** full-splice match (FSM), **F** incomplete-splice match (ISM), **G** novel in catalog (NIC), and **H** novel not in catalog (NNC). Each transcript isoform is represented by a single row, with bars corresponding to DOK (blue), DOK-AL (green), and DOK-NNKL (red), allowing direct comparison of transcript abundance across the three experimental groups. Compared with parental DOK cells, carcinogen-adapted cells exhibited an increased proportion of NIC and NNC transcript isoforms. **I** RNA-seq analysis of DOK cells following 12 months of exposure to NNK or arecoline revealed global transcriptome reprogramming, including the induction of multiple lncRNAs. **J** RNA-seq coverage tracks showing increased expression of *RP11-30P6.6* and *RP11-114H23.1* in NNK-L and Are-L cells relative to that in the parental DOK. **K**–**L** qRT-PCR validation of *RP11-30P6.6* expression in DOK cells after long-term (12 months) or short-term (72 h) exposure to NNK or arecoline. The expression levels were normalized to those of *GAPDH*. *n* = 3. Data are presented as the mean ± SD of values from at least three independent experiments. Statistical significance was determined using a two-tailed unpaired Student’s *t*-test or one-way ANOVA with appropriate post hoc tests; *P < 0.05, **P < 0.01, ***P < 0.001

Based on our previous reports, EGFRvIII was detectable in a substantial fraction of head and neck cancers by immunohistochemistry, with EGFRvIII-specific junctions confirmed via Sanger sequencing in OSCC [3, 41]. Consistent with these observations, western blotting analysis revealed higher EGFRvIII expression in most OSCC cell lines than in DOK (Fig. 1A). To test whether tobacco- and areca-derived carcinogens modulate this axis, two cell lines, DOK-NNKL and DOK-AL, were established by continuously exposing them to noncytotoxic NNK (10 μM) and arecoline (50 μM), respectively, for 12 months. As shown in Fig. 1B, EGFRvIII expression in these two cell lines was higher than that in DOK cells. In addition, the expression levels of EGFRvIII increased after treatment with NNK and arecoline for 72 h (Fig. 1C). Taken together, both long- and short-term NNK/arecoline treatment enhanced EGFR/EGFRvIII expression in oral epithelial cell models. Two GBM cell lines, U87 (EGFRvIII-low) and A172 (EGFRvIII-high), with different expression levels of EGFRvIII, were also analyzed (Fig. 1B). The expression levels of EGFRvIII in both these lines increased after treatment with NNK and arecoline for 96 h (Figure S1).

### Carcinogen-induced remodeling of *EGFR* isoform architecture detected using PacBio long-read sequencing

To obtain isoform-resolved evidence for altered *EGFR* processing, we performed PacBio circular consensus (CCS) amplicon sequencing on DOK, DOK-AL, and DOK-NNKL cells. CCS reads were aligned to the *EGFR* reference and classified as full-splice match (FSM), incomplete-splice match (ISM), novel in catalog (NIC) or novel not in catalog (NNC). Compared with DOK cells, which exhibited a predominance of FSM/ISM, both DOK-AL and DOK-NNKL cells showed a pronounced shift in isoform composition with enrichment of NIC/NNC isoforms (Fig. 1D–H). Long-read transcript annotation identified remodeling of the *EGFR* transcript architecture in DOK-AL and DOK-NNKL cells, including changes in transcript isoform composition and splice-junction patterns. Together, these transcript-level alterations are consistent with altered *EGFR* transcript processing associated with *EGFRvIII* expression. These observations are further supported by RT-PCR, Sanger sequencing, immunoblotting, and EGFR minigene assays **(**Figs. 1 and 4). Among the transcript isoforms identified using PacBio Iso-Seq, *PB.11430.142*, an ISM-classified transcript, showed the highest predicted protein sequence similarity to canonical *EGFRvIII* among the detected transcript isoforms and exhibited a marked increase in DOK-AL and DOK-NNKL cells compared with parental DOK cells. Figure S2 provides detailed structural schematics and sequence information for representative *EGFR* splice isoforms, including the exon 1–exon 8 junction characteristic of canonical *EGFRvIII* and the PacBio Iso-Seq-derived transcript *PB.11430.142*. Together, these findings provide transcript-level evidence of carcinogen-associated remodeling of the *EGFR* isoform landscape.

**Fig. 2:**
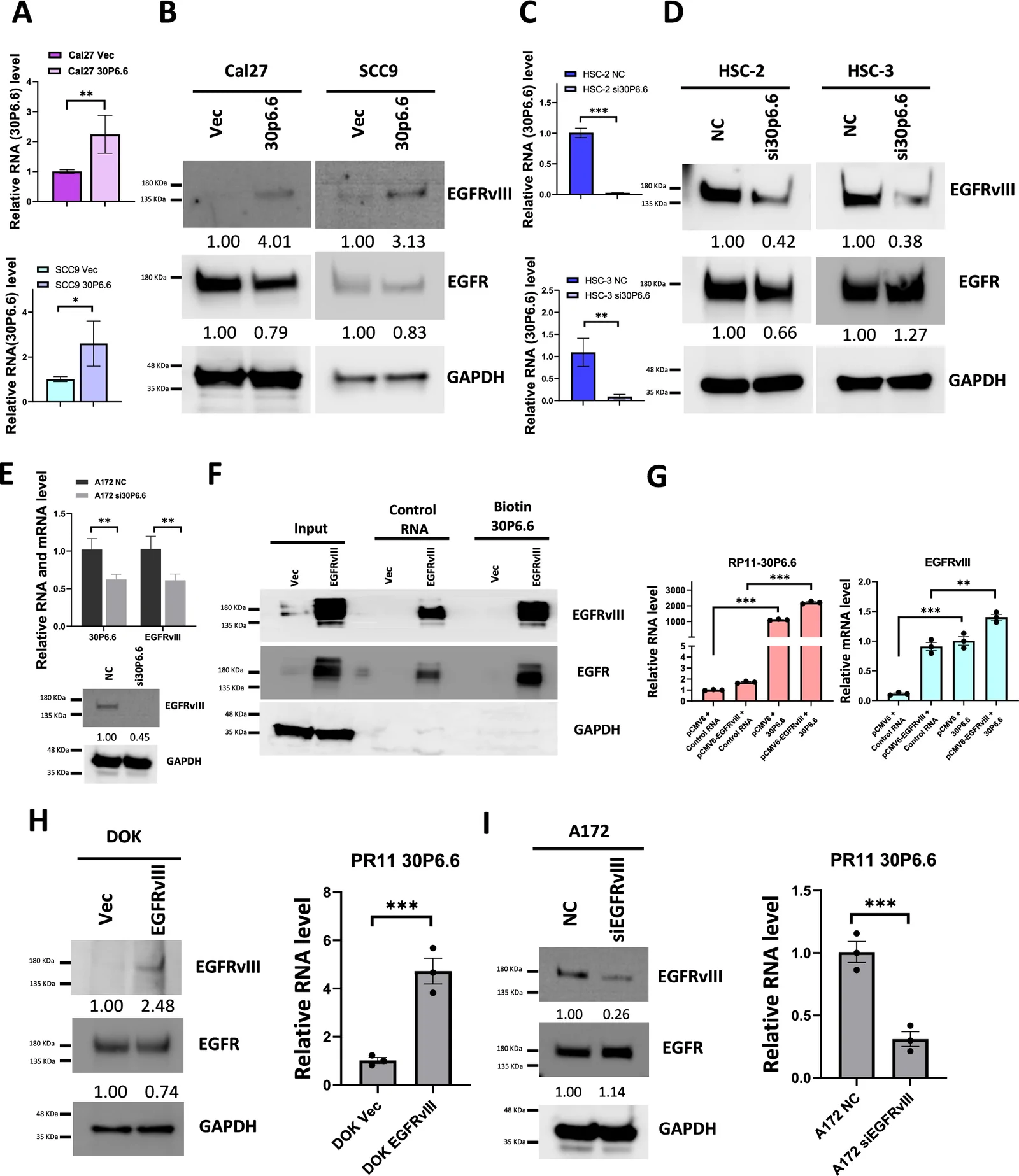
RP11-30P6.6 regulates EGFRvIII expression in OSCC and glioblastoma cell lines. **A** qRT-PCR analysis of *RP11-30P6.6* expression in Cal-27 and SCC-9 cells transfected with *RP11-30P6.6* or empty vector for 72 h. *n* = 3. **B** Immunoblot analysis of EGFR and EGFRvIII in the same samples. GAPDH was used as a loading control. Densitometric quantification normalized to GAPDH is shown beneath the blots. *n* = 3. **C** qRT-PCR analysis of *RP11-30P6.6* expression in HSC-2 and HSC-3 cells transfected with siRP11-30P6.6 or negative control (NC) for 72 h. *n* = 3. **D** Immunoblot analysis of EGFR and EGFRvIII in the corresponding samples. GAPDH was used as a loading control. Densitometric quantification was performed as in (**B**). *n* = 3. **E** Top: qRT-PCR analysis of *RP11-30P6.6* and *EGFRvIII* transcript levels in A172 cells transfected with siRP11-30P6.6 or NC for 72 h. Bottom: immunoblot analysis of EGFR and EGFRvIII proteins in the same samples. GAPDH was used as a loading control. *n* = 3. **F** Biotinylated RNA pull-down assay using *RP11-30P6.6*. Biotin-labeled *RP11-30P6.6* or control RNA was incubated with cell lysates, followed by capture with streptavidin beads. Immunoblot analysis showed preferential recovery of EGFRvIII in the *RP11-30P6.6* pull-down fraction relative to the unrelated RNA control. Control RNA and input lysates served as negative and positive controls, respectively. *n* = 3. **G** Biotinylated RNA pull-down assay using *RP11-30P6.6*. Biotin-labeled *RP11-30P6.6* or control RNA was incubated with cell lysates, followed by capture with streptavidin beads. qRT-PCR analysis showed preferential recovery/enrichment of the indicated transcripts in the *RP11-30P6.6* pull-down fraction relative to control RNA. *n* = 3. **H** Left: immunoblot analysis of EGFR and EGFRvIII in DOK cells transfected with EGFRvIII or vector control. Right: qRT-PCR analysis of *RP11-30P6.6* expression in the same samples. Expression levels were normalized to those of *GAPDH*. *n* = 3. **I** Left: immunoblot analysis of EGFR and EGFRvIII in A172 cells transfected with siEGFRvIII or NC. Right: qRT-PCR analysis of *RP11-30P6.6* and *EGFRvIII* transcripts in the same samples. *GAPDH* was used as a loading control and normalization control. *n* = 3. Data are presented as the mean ± SD of values from three independent experiments. Statistical significance was determined using a two-tailed unpaired Student’s *t*-test (two groups) or one-way ANOVA with appropriate post-hoc tests (≥ 3 groups); *P < 0.05, **P < 0.01, ***P < 0.001

### Carcinogen exposure increases lncRNA expression

We next examined global transcriptomic changes in DOK, DOK-AL, and DOK-NNKL cells using RNA-seq. Both NNK and arecoline induced widespread changes in gene expression. *RP11-30P6.6* and *RP11-114H23.1* were initially identified as lncRNA candidates from the RNA-seq screen. *RP11-30P6.6* was consistently and robustly upregulated in both DOK-NNKL and AL cells (Fig. 1I, J). qRT-PCR analysis further confirmed that *RP11-30P6.6* expression increased in DOK cells after both long- and short-term exposure to NNK or arecoline (Fig. 1K, L). In contrast, *RP11-114H23.1* was not prioritized for functional studies because its induction was less consistent across validation conditions. These findings indicate that carcinogen exposure reshapes the cellular transcriptome and induces selected lncRNAs, among which *RP11-30P6.6* was selected for subsequent functional investigations.

To define the functional role of *RP11-30P6.6*, we performed gain- and loss-of-function experiments using OSCC models. Stable overexpression of *RP11-30P6.6* in Cal-27 and SCC-9 cells caused an increase in EGFRvIII protein expression (Fig. 2A, B) and enhanced cellular proliferation, clonogenic growth, and Transwell migration (Figure S3A, C, E). In contrast, siRNA-mediated knockdown of *RP11-30P6.6* in HSC-2 and HSC-3 cells decreased the EGFRvIII protein expression (Fig. 2C, D) and suppressed cellular proliferation, colony formation, and migration (Figure S3B, D, F). Consistently, siRP11-30P6.6 decreased EGFRvIII expression in DOK, DOK-AL, and DOK-NNKL cells (Figure S4A, B). Similar results were obtained in the GBM cell line A172, suggesting that this regulatory relationship is not restricted to OSCC cells (Fig. 2E). Collectively, these findings indicate that *RP11-30P6.6* is a carcinogen-inducible oncogenic lncRNA that promotes proliferation and invasiveness of OSCC cells.

To examine whether *RP11-30P6.6* was associated with *EGFRvIII*-related RNA and proteins under the RNA pull-down conditions, we performed biotinylated RNA pull-down assays using full-length *RP11-30P6.6*. *EGFRvIII* transcripts and associated protein signals were preferentially recovered in the *RP11-30P6.6* pull-down relative to the unrelated RNA control **(**Fig. 2F, G**)**. These findings support a biochemical association with RP11-30P6.6-associated pull-down fractions but do not establish direct molecular binding or formation of a defined RNP complex. Furthermore, *EGFRvIII* overexpression in DOK cells led to increased *RP11-30P6.6* expression and vice versa in A172 cells **(**Fig. 2H, I**)**. Taken together, our data demonstrate preferential recovery of EGFRvIII-related RNA/protein signals in *RP11-30P6.6*-associated pull-down fractions and support an association between *RP11-30P6.6* and altered EGFRvIII expression.

### NNK and arecoline reprogram RNA splicing regulators to promote EGFRvIII expression

**Fig. 3:**
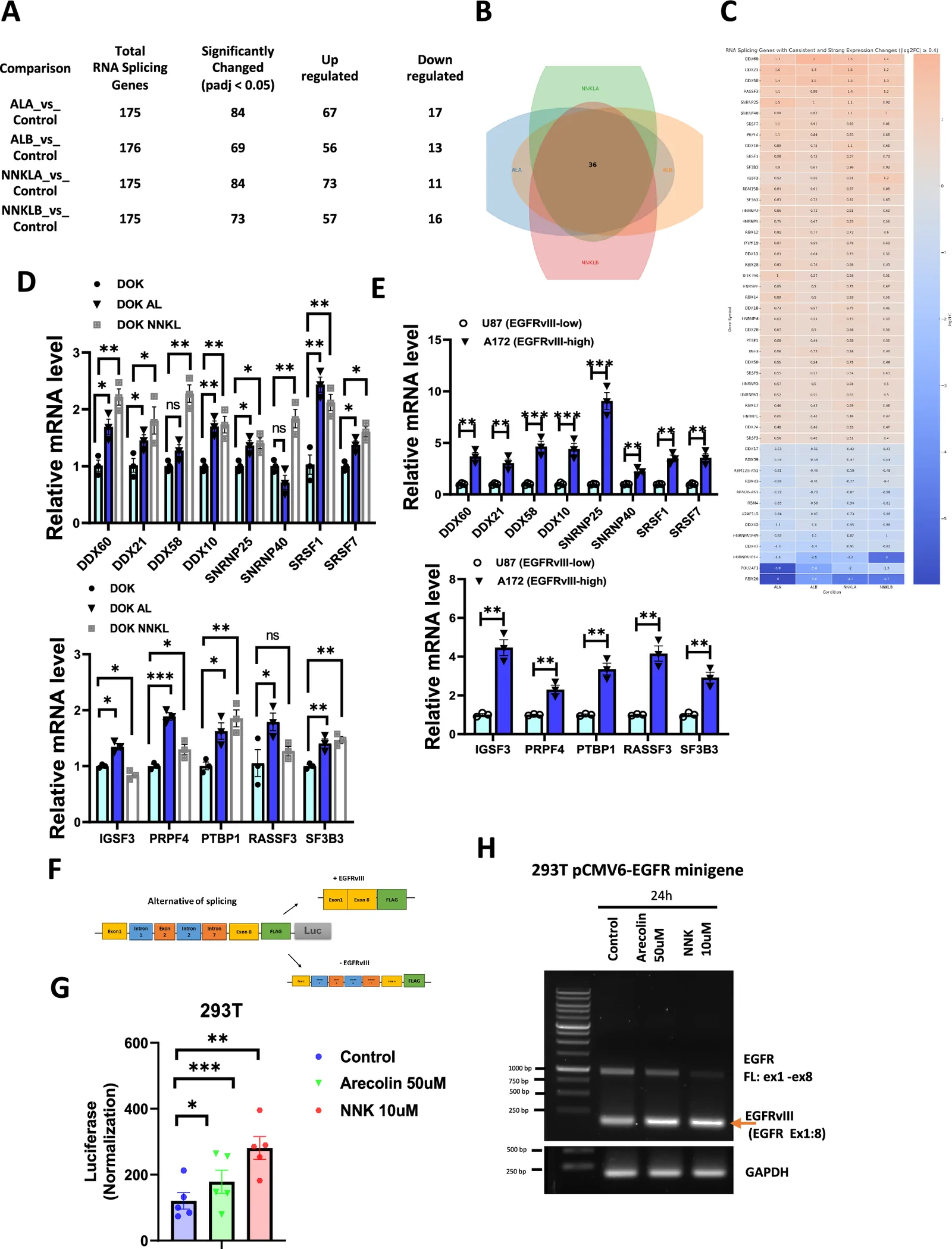
NNK and arecoline reprogram splicing regulators to promote EGFRvIII formation. **A** RNA-seq analysis of representative splicing regulators in DOK-AL and DOK-NNKL cells, presented as log2 fold change relative to parental DOK cells. Samples A and B denote biological replicates. **B** Venn diagram integrating two comparisons (DOK-AL vs. DOK and DOK-NNKL vs. DOK), identifying 36 splicing-related genes that were consistently dysregulated in both models. **C** Heat map showing log_2_ fold changes in the 36 shared splicing regulators (red, upregulated; blue, downregulated). **D** qRT-PCR validation of selected splicing regulators in DOK, DOK-AL, and DOK-NNKL cells. Expression levels were normalized to those *GAPDH* and are presented relative to those in DOK cells. *n* = 3. **E** qRT-PCR validation of the same regulators in A172 and U87 glioblastoma cells. Expression levels were normalized to those of *GAPDH* and are presented relative to those in U87 cells. *n* = 3. **F** Schematic representation of the *EGFR* minigene reporter construct used to monitor exon skipping. **G** Luciferase reporter assay in 293 T cells cotransfected with the *EGFR* minigene and treated with NNK and/or arecoline, indicating increased reporter activity under carcinogen exposure. *n* = 3. **H** RT-PCR analysis of *EGFR* minigene-derived transcripts. Representative agarose gels showing induction of *EGFRvIII*-specific (exons 1:8) amplicons following NNK and/or arecoline treatment, with junction identity confirmed via Sanger sequencing. *n* = 3. Data are presented as the mean ± SD of values from three independent experiments. Statistical significance was determined using a two-tailed unpaired Student’s *t*-test (two groups) or one-way ANOVA with appropriate post-hoc tests (≥ 3 groups); *P < 0.05, **P < 0.01, ***P < 0.001

In recent studies, lncRNAs have been suggested to regulate alternative splicing through interactions with pre-mRNAs and splicing factors, for example, via modulation of spliceosome activity and exon selection [40]. To investigate whether *RP11-30P6.6* contributes to *EGFRvIII* generation through splicing regulation, we analyzed RNA-seq profiles from DOK, DOK-AL, and DOK-NNKL cells. RNA-seq analysis identified 36 splicing- and RNA-processing–related genes that were altered in DOK-AL and DOK-NNKL cells compared with those in parental DOK cells (Fig. 3A–C). Among these genes, several RNA-processing regulators, including *SRSF1*, *SRSF7*, *DDX21*, *DDX60*, and *hnRNPA1*, exhibited condition-dependent changes in carcinogen-exposed cells. The change in expression of these genes was validated via qRT-PCR in DOK, DOK-AL, and DOK-NNKL cells, and similar patterns were observed in GBM cells (Fig. 3D, E).

To determine whether carcinogen exposure promoted *EGFRvIII* splicing, we generated an *EGFR* minigene reporter containing exon 1:8 with partial intronic sequences to preserve key *cis*-regulatory elements (Fig. 3F). This design allowed for the detection of exon 1:8 junction formation, corresponding to the skipping of exons 2–7. In 293 T cells, treatment with NNK or arecoline significantly increased the reporter activity and enhanced the formation of the exon 1:8 splice junction, suggesting increased *EGFRvIII* expression (Fig. 3G). RT-PCR analysis further confirmed the presence of *EGFRvIII*-specific amplicons, and Sanger sequencing verified the accurate exon 1:8 junction sequence (Fig. 3H, data not shown).

We then performed siRNA-mediated knockdown of selected splicing regulators to identify the potential functional drivers of *EGFRvIII* expression. RT-PCR analysis demonstrated that depletion of *PTBP1*, *SRSF1*, and *SRSF7* decreased the *EGFRvIII* abundance in 293 T cells expressing the *EGFR* minigene. Moreover, depletion of *SRSF1* consistently reduced endogenous EGFRvIII protein levels in DOK-AL, DOK-NNKL, and HSC-3/M3 cells. Notably, siRNA knockdown of *RP11-30P6.6* led to a reduction in the expression of SRSF1 instead of SRSF7, DDX21, DDX60, and PTBP1 (Figure S5A-4C). Taken together, these data suggest that SRSF1 is a major functional mediator associated with *RP11-30P6.6-*dependent regulation of EGFRvIII expression.

### SRSF1 and RP11-30P6.6 coordinate an ncRNA–splicing module in regulating EGFRvIII expression

**Fig. 4:**
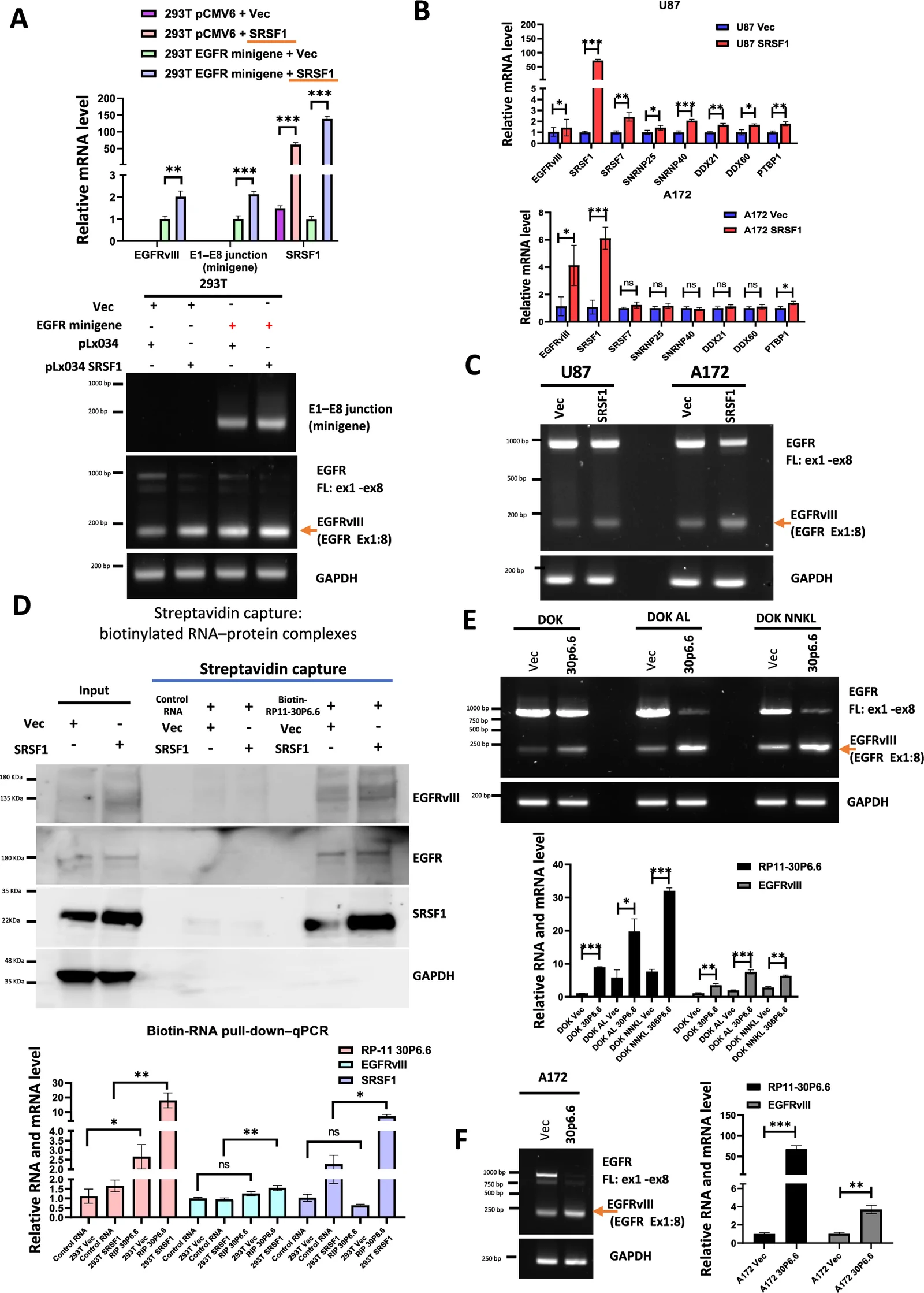
SRSF1 promotes *EGFRvIII* expression and increases exon 1–exon 8 junction-containing transcripts. **A** qRT-PCR analysis of SRSF1, EGFRvIII, and minigene-derived exon 1–exon 8 junction transcripts, together with RT-PCR analysis of products obtained in the pCMV6-EGFR minigene experiment, in 293 T cells cotransfected with pLX304 (vector) or pLX304-SRSF1 for 48 h. Minigene-specific RT-PCR and qRT-PCR were performed using an exon 1–exon 8 junction-specific forward primer and an exon 8–FLAG junction-specific reverse primer. Representative agarose gels show the minigene-derived exon 1–exon 8 junction product, with junction identity verified by Sanger sequencing. Data are presented as mean ± SD from three independent experiments (n = 3). **B** qRT-PCR analysis of *EGFRvIII*, *SRSF1*, and splicing-associated factors (*SRSF7*, *SNRNP25*, *SNRNP40*, *DDX21*, *DDX60*, and *PTBP1*) in U87 and A172 cells transfected with pLx304 or pLx304-*SRSF1* for 48 h. Expression levels were normalized to those of *GAPDH*. *n* = 3. **C** RT-PCR analysis of endogenous *EGFR* transcripts in U87 and A172 cells transfected with pLx304 or pLx304-*SRSF1* for 48 h. Representative agarose gels show the *EGFRvIII*-specific exon 1 → 8 amplicon following *SRSF1* overexpression. *n* = 3. **D** Biotinylated RNA pull-down assay using in vitro-transcribed (IVT) *RP11-30P6.6* RNA or an unrelated control RNA. 293 T cells were transfected with an SRSF1 expression plasmid or the corresponding vector control and lysed 24 h after transfection. Cell lysates were incubated with biotinylated *RP11-30P6.6* RNA or unrelated control RNA, followed by streptavidin-mediated capture. Immunoblot analysis showed preferential recovery of SRSF1 in the *RP11-30P6.6* RNA pull-down relative to the unrelated RNA control under both vector-control and SRSF1-overexpressing conditions. qRT-PCR analysis was performed on RNA recovered from the pull-down fractions. *n* = 3. **E** Top: RT-PCR analysis of endogenous *EGFR* transcripts in DOK, DOK-AL, and DOK-NNKL cells transfected for 24 h with empty vector (pCMV6) or *RP11-30P6.6*-expressing plasmid (pCMV6-*RP11-30P6.6*). Representative agarose gels show the *EGFRvIII*-specific exon 1 → 8 amplicon under the indicated conditions. Bottom: qRT-PCR analysis of *RP11-30P6.6* and *EGFRvIII* expression in the same samples. Expression levels were normalized to those of *GAPDH*. *n* = 3. **F** Left: RT-PCR analysis of endogenous *EGFR* transcripts in A172 cells transfected for 24 h with negative-control siRNA (NC), *RP11-30P6.6* siRNA (si-*RP11-30P6.6*), empty vector (pCMV6), or *RP11-30P6.6*-expressing plasmid (pCMV6-*RP11-30P6.6*). Representative agarose gels show the *EGFRvIII*-specific exon 1 → 8 amplicon under the indicated conditions. Right: qRT-PCR analysis of *RP11-30P6.6* and *EGFRvIII* expression in the same samples. Expression levels were normalized to those of *GAPDH*. *n* = 3. Data are presented as the mean ± SD from three independent experiments. Statistical significance was determined using a two-tailed unpaired Student’s *t*-test for comparisons between two groups or one-way ANOVA followed by an appropriate post hoc multiple-comparison test for comparisons among three or more groups; *P < 0.05, **P < 0.01, and ***P < 0.001

We further used an EGFR minigene assay to examine the effect of SRSF1 on minigene-derived exon 1–exon 8 junction transcripts. Minigene-specific RT-PCR detected the expected exon 1–exon 8 junction product in EGFR minigene-transfected cells but not in empty-vector controls, and the identity of this product was confirmed by Sanger sequencing **(**Fig. 4A**)**. Minigene-specific qRT-PCR further showed that SRSF1 overexpression significantly increased the abundance of the minigene-derived exon 1–exon 8 junction transcript compared with the corresponding minigene plus vector control (Fig. 4A). Consistently, SRSF1 overexpression significantly increased endogenous EGFRvIII transcript levels in A172 cells. Endogenous EGFRvIII transcript abundance was quantified by qRT-PCR **(**Fig. 4B**)**, whereas the exon 1 → 8 junction was independently detected by RT-PCR **(**Fig. 4C**)**. Next, we examined whether SRSF1 was preferentially recovered in RP11-30P6.6-associated pull-down fractions. Biotinylated RNA pull-down assays using in vitro-transcribed *RP11-30P6.6* RNA revealed preferential recovery of SRSF1 relative to the control RNA, supporting an association between *RP11-30P6.6* and SRSF1-containing pull-down fractions **(**Fig. 4D**)**. We further evaluated the functional role of RP11-30P6.6 in EGFRvIII splicing. Overexpression of *RP11-30P6.6* in DOK, DOK-AL, and DOK-NNKL cells increased the abundance of *EGFRvIII* transcripts (Fig. 4E). Similar results were obtained in A172 cells (Fig. 4F). Together, these results indicate that RP11-30P6.6 is associated with EGFRvIII-related RNA processing and SRSF1-containing pull-down fractions, consistent with a model in which RP11-30P6.6 may contribute to SRSF1-mediated regulation of EGFRvIII splicing.

**Fig. 5:**
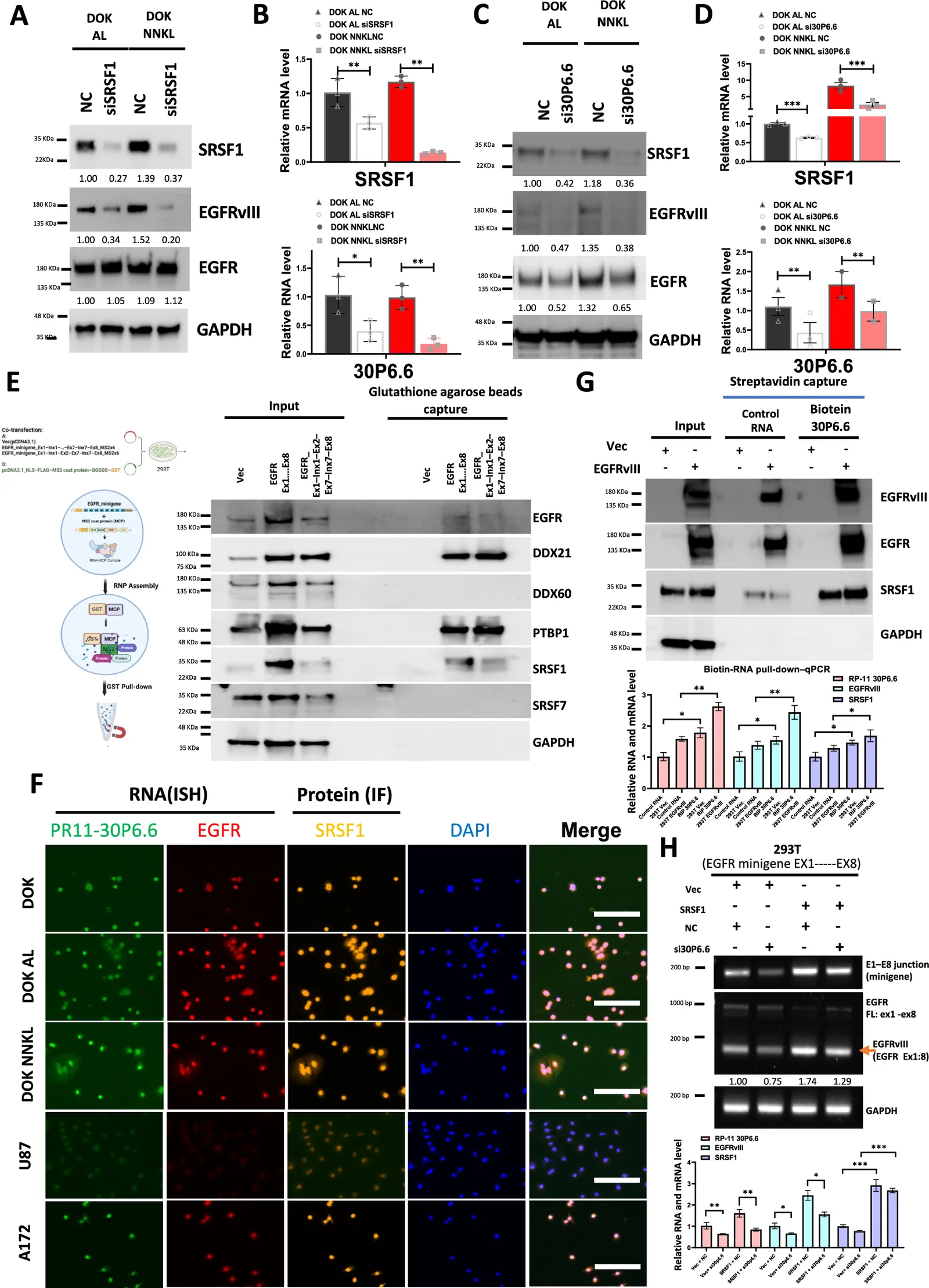
NNK and arecoline drive *EGFRvIII* splicing through a SRSF1–*RP11-30P6.6* regulatory axis. **A** Immunoblot analysis of EGFR, EGFRvIII, and splicing-associated factors (including SRSF1) in DOK-AL and DOK-NNKL cells transfected with siSRSF1 or negative control (NC) for 72 h. GAPDH was used as a loading control. Densitometric quantification normalized to GAPDH is shown beneath the blots. *n* = 3. **B** qRT-PCR analysis of *RP11-30P6.6* and *SRSF1* in DOK, DOK-AL, and DOK-NNKL cells transfected with siSRSF1 or NC for 72 h. Expression levels were normalized to those of *GAPDH*. *n* = 3. **C** Immunoblot analysis of EGFR, EGFRvIII, and splicing-associated factors (including SRSF1) in DOK, DOK-AL, and DOK-NNKL cells transfected with siRP11-30P6.6 or NC for 72 h. GAPDH was used as a loading control. Densitometric quantification normalized to GAPDH is shown beneath the blots. *n* = 3. **D** qRT-PCR analysis of *SRSF1* and *RP11-30P6.6* in DOK, DOK-AL, and DOK-NNKL cells transfected with siRP11-30P6.6 or NC for 72 h. Expression levels were normalized to those of *GAPDH*. *n* = 3. **E** Schematic of the MS2–MCP RNA affinity purification workflow. 293 T cells were cotransfected with an *EGFR* minigene containing MS2 hairpin repeats and an MCP–GST fusion protein. MS2-mediated RNA-associated ribonucleoprotein (RNP) complexes were isolated using glutathione agarose bead-based GST pull-down. Immunoblot analysis of proteins recovered in the GST pull-down fraction is shown. SRSF1, PTBP1, and DDX21 were detected in the GST pull-down fraction, consistent with their association with *EGFR* minigene-derived RNP complexes. Input and post-pull-down supernatant fractions are shown as controls. *n* = 3. **F** RNA in situ hybridization combined with immunofluorescence showing *RP11-30P6.6* (green), *EGFR* (red), and *SRSF1* (yellow). Partial colocalization is observed in nuclear puncta. Nuclei were counterstained with DAPI (blue). Scale bars, 150 μm. *n* = 2. **G** Biotinylated RNA pull-down assay using in vitro-transcribed (IVT) *RP11-30P6.6* RNA or an unrelated control RNA. Cell lysates from vector-control or *EGFRvIII*-overexpressing cells were incubated with biotinylated *RP11-30P6.6* RNA or unrelated control RNA, followed by streptavidin-mediated capture. Immunoblot analysis showed greater recovery of SRSF1 with the *RP11-30P6.6* RNA than with the unrelated control RNA under both vector-control and *EGFRvIII*-overexpressing conditions. *n* = 3. **H** *EGFR* minigene splicing assay in 293 T cells cotransfected with the indicated siRNAs and/or expression constructs and harvested 24 h post-transfection. Representative agarose gel showing modulation of *EGFRvIII*-specific (E1 → E8) splicing. qRT-PCR analysis of *RP11-30P6.6* and *SRSF1* transcripts was performed in parallel. *n* = 3. Data are presented as mean ± SD of values from three independent experiments, unless otherwise indicated. Statistical significance was determined using an unpaired two-tailed Student’s *t*-test (two groups) or one-way ANOVA (≥ 3 groups)

Given the coordinated induction of splicing factors and *RP11-30P6.6* following carcinogen exposure, we investigated whether these regulators function cooperatively to control *EGFRvIII* expression. In DOK-AL and DOK-NNKL cells, *SRSF1* knockdown markedly reduced EGFRvIII protein levels and was accompanied by decreased *RP11-30P6.6* expression (Fig. 5A, B). In contrast, depletion of *RP11-30P6.6* reduced SRSF1 protein levels and concomitantly decreased *EGFRvIII* expression (Fig. 5C, D), indicating reciprocal regulatory interactions between these components. To further examine RNA-processing factors recovered with EGFR minigene-derived RNA, we performed MS2-based RNA affinity purification. 293 T cells were cotransfected with an *EGFR* minigene harboring MS2 hairpin repeats and an MCP–GST fusion construct, enabling selective capture of the RNA-bound protein complex. GST pull-down followed by immunoblotting revealed preferential recovery of multiple RNA-processing factors with the MS2-tagged *EGFR* minigene RNA. Notably, SRSF1 was robustly enriched in the recovered fraction, supporting its association with *EGFR* minigene RNA-containing RNP fractions. Additional splicing regulators, including PTBP1 and DDX21, were also detected. These findings provide biochemical evidence that *EGFR* minigene-derived RNA is recovered together with several RNA-processing factors under the MS2 affinity-purification conditions (Fig. 5E). Immunofluorescence analysis revealed nuclear-enriched staining patterns for SRSF1 consistent with RNA-processing compartments. Consistently, RNA in situ hybridization combined with immunofluorescence revealed *RP11-30P6.6* signals in nuclear-enriched regions, in close spatial proximity to SRSF1-positive RNA-processing compartments (Fig. 5F), supporting their spatial proximity within RNA-processing assemblies. Furthermore, RNA pull-down assays using biotinylated *RP11-30P6.6* revealed the enrichment of both SRSF1 protein and *EGFRvIII* transcripts in the pull-down fraction (Fig. 5G), suggesting preferential recovery of SRSF1 protein and EGFRvIII transcripts in RP11-30P6.6-associated pull-down fractions.

Functionally, *EGFR* minigene assays in 293 T cells showed that *SRSF1* overexpression increased the abundance of the minigene-derived exon 1–exon 8 junction transcript, whereas this effect was attenuated upon *RP11-30P6.6* depletion (Fig. 5H), indicating that *RP11-30P6.6* may contribute to the SRSF1-mediated regulation of EGFRvIII splicing. Collectively, these data indicate that SRSF1 and *RP11-30P6.6* are functionally associated with *EGFRvIII*-related regulation of RNA. These findings uncover a carcinogen-responsive ncRNA–splicing interface linking post-transcriptional regulation with spliceosomal control of the expression of the oncogenic isoform. Furthermore, they indicate that *RP11-30P6.6* may contribute to an RNA-processing environment permissive for *EGFRvIII*-associated transcript output.

### Identification and functional evaluation of candidate miRNAs showing differential responsiveness between *EGFRvII*I and WT-*EGFR*

**Fig. 6:**
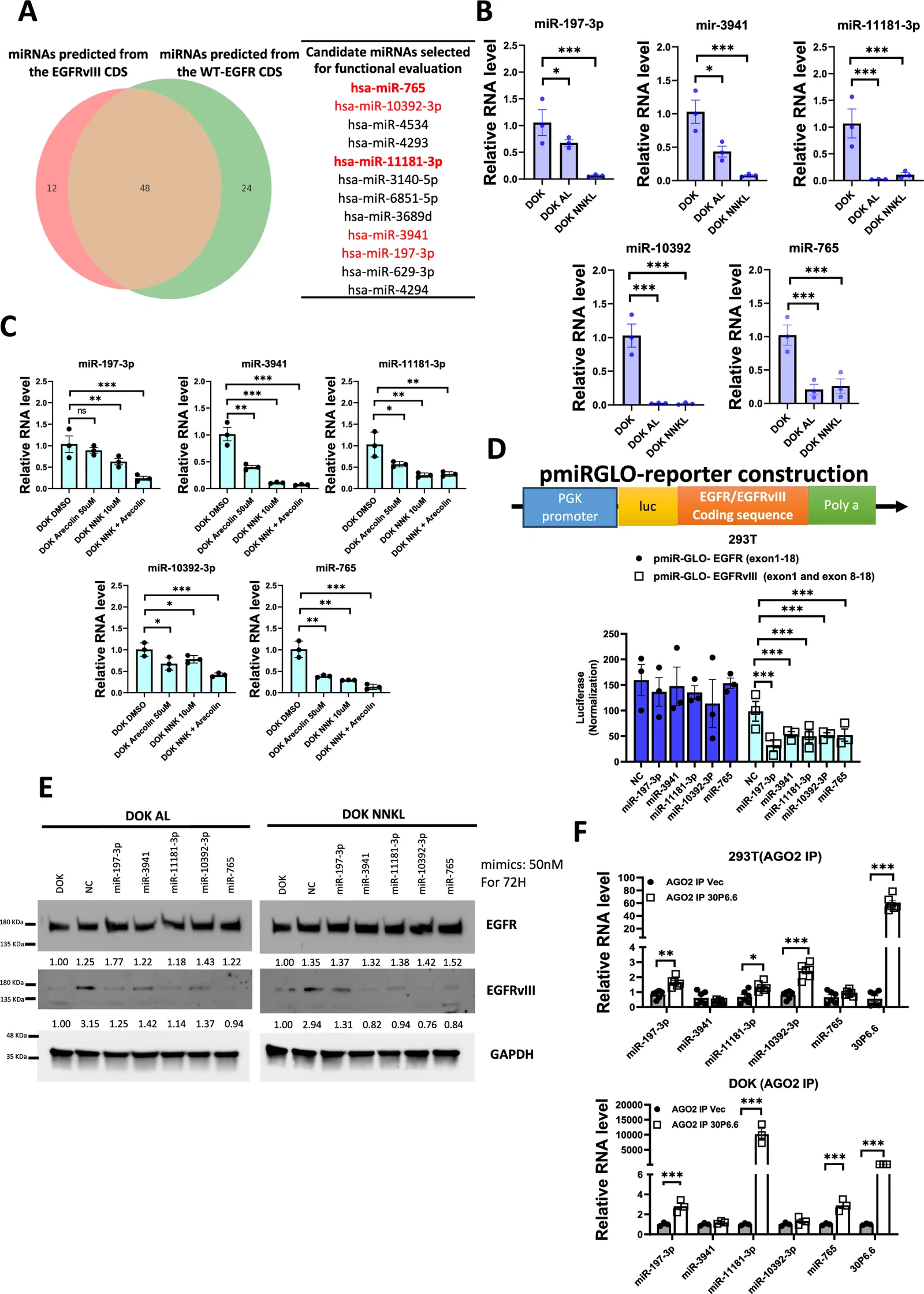
*RP11-30P6.6*–associated miRNAs show differential regulatory effects on *EGFRvIII* and WT-*EGFR.* **A** Venn diagram comparing miRDB Custom Prediction outputs obtained by independently analyzing the complete EGFRvIII and WT-EGFR coding sequences (CDSs). Twelve miRNAs were identified in the EGFRvIII prediction output but not in the WT-EGFR prediction output. Among these 12 miRNAs, five candidates—miR-197-3p, miR-11181-3p, miR-765, miR-10392-3p, and miR-3941—were selected for subsequent functional analysis. **B** qRT-PCR analysis of the indicated miRNAs in DOK cells chronically exposed to nicotine-derived nitrosamine ketone (NNK) (10 µM) or arecoline (50 µM) for 12 months. Expression levels were normalized to U6 and compared with those in parental DOK cells. *n* = 3. **C** qRT-PCR analysis of the same miRNAs in DOK cells treated with NNK (10 µM), arecoline (50 µM), or both for 96 h. Expression levels were normalized to *U6*. *n* = 3. **D** Luciferase reporter assays in 293 T cells cotransfected with pmiRGLO vectors carrying either WT-*EGFR* CDS or *EGFRvIII* CDS together with the indicated miRNA mimics. Relative luciferase activity was measured 48 h post-transfection and normalized to the Renilla luciferase activity, demonstrating preferential repression of *EGFRvIII* over WT-*EGFR*. *n* = 3. **E** Immunoblot analysis of EGFR and EGFRvIII in DOK-AL and DOK-NNKL cells transfected with the indicated miRNA mimics for 72 h. GAPDH was used as a loading control. Densitometric quantification normalized to GAPDH is shown beneath the blots. *n* = 3. **F** AGO2 immunoprecipitation in 293 T and DOK cells overexpressing *RP11-30P6.6* or vector control, followed by qRT-PCR analysis of associated miRNAs (miR-197-3p, miR-11181-3p, miR-765, miR-10392-3p, and miR-3941), indicating enhanced association of the tested miRNAs with AGO2-containing complexes. *n* = 3. Data are presented as the mean ± SD of values from three independent experiments. Statistical significance was determined using a two-tailed unpaired Student’s *t*-test (two groups) or one-way ANOVA with appropriate post-hoc tests (≥ 3 groups); *P < 0.05, **P < 0.01, ***P < 0.001

To elucidate the mechanism by which *RP11-30P6.6* regulates *EGFRvIII* expression, we used miRDB Custom Prediction (https://mirdb.org) to identify candidate miRNAs using the complete coding sequences (CDSs) of *EGFRvIII* and WT-*EGFR* as independent target sequences. Comparison of the prediction outputs identified 48 miRNAs shared between the two outputs and 12 miRNAs identified in the *EGFRvIII* prediction output but not in the WT-*EGFR* prediction output. Of these 12 miRNAs, five candidates (hsa-miR-10392-3p, hsa-miR-3941, hsa-miR-197-3p, hsa-miR-11181-3p, and hsa-miR-765) were selected for further functional analysis. Of these, hsa-miR-11181-3p and hsa-miR-765 were additionally predicted to bind *RP11-30P6.6*, suggesting their involvement in *RP11-30P6.6*-mediated regulation of *EGFRvIII* expression (Fig. 6A and Figure S7). We further confirmed that these miRNAs were consistently downregulated in DOK-NNKL and DOK-AL cells and in DOK cells treated with NNK, arecoline, or both for 96 h (Fig. 6B, C).

To functionally compare miRNA responsiveness between the two *EGFR* isoforms, we cloned the full-length *EGFRvIII* CDS into the pmirGLO luciferase reporter vector. A parallel construct carrying the full-length WT-*EGFR* CDS was generated for comparison. This design allowed direct comparison of miRNA-mediated regulation of the WT-*EGFR* and *EGFRvIII* CDSs under the same experimental conditions. The luciferase reporter assays showed that the five tested miRNAs significantly suppressed the *EGFRvIII* reporter, whereas the WT-*EGFR* reporter did not exhibit the same degree of suppression (Fig. 6D). Transfection of these miRNA mimics also reduced endogenous EGFRvIII protein levels in DOK-AL and DOK-NNKL cells, whereas WT-EGFR protein levels showed comparatively smaller changes under the same conditions (Fig. 6E).

To determine whether restoration of candidate miRNAs could counteract *RP11-30P6.6*-associated EGFRvIII expression, HSC-3 cells were co-transfected with either an *RP11-30P6.6* expression plasmid or a vector control together with individual candidate miRNA mimics. qRT-PCR confirmed successful overexpression of the respective miRNAs and sustained *RP11-30P6.6* overexpression following co-transfection. Immunoblot analysis showed that miR-197-3p, miR-3941, and miR-11181-3p reduced EGFRvIII protein expression in vector-control cells. Importantly, when the same miRNA mimic was compared between vector-control and *RP11-30P6.6*-overexpressing cells, EGFRvIII protein levels remained higher in the *RP11-30P6.6*-overexpressing cells. These results indicate that restoration of these miRNAs suppresses EGFRvIII expression, whereas elevated *RP11-30P6.6* partially attenuated this suppression (Figure S8A, B). These findings provide additional functional evidence consistent with a ceRNA-like relationship between *RP11-30P6.6* and the tested miRNAs. Furthermore, siRNA-mediated knockdown of *RP11-30P6.6* increased the levels of these five miRNAs in HSC-3 cells, whereas the opposite pattern was observed following *RP11-30P6.6* overexpression in SCC-9 cells (Figure S9A, B).

AGO2 immunoprecipitation in *RP11-30P6.6*-overexpressing 293 T and DOK cells revealed increased enrichment of four tested miRNAs (miR-197-3p, miR-11181-3p, miR-765, and miR-10392-3p) compared with controls, with the most pronounced enrichment observed for miR-197-3p, miR-11181-3p, and miR-765 (Fig. 6F). Given that AGO2 is a core component of the RNA-induced silencing complex (RISC), these findings are consistent with an association of these miRNAs with AGO2-containing complexes and support a model in which *RP11-30P6.6* may modulate their availability or activity. Taken together, these results are consistent with, but do not by themselves establish, a ceRNA-like mechanism involving *RP11-30P6.6* and miRNAs capable of regulating *EGFRvIII* expression.

To determine whether EGFRvIII may in turn modulate this ncRNA network, we assessed *RP11-30P6.6* and miRNA expression following *EGFRvIII* overexpression. In Cal-27 cells, enforced *EGFRvIII* expression increased *RP11-30P6.6* levels and concomitantly reduced the expression of multiple tested miRNAs (Figure S10A–C). Similar changes were observed in U87 and A172 cells (Figure S10D). These coordinated changes indicate that *EGFRvIII* overexpression is accompanied by reciprocal changes in components of this ncRNA regulatory network. The inverse relationship between *RP11-30P6.6* and the tested miRNAs is consistent with a model in which *RP11-30P6.6* may attenuate miRNA-mediated repression and thereby contribute to the maintenance of *EGFRvIII* expression. Together, these observations are consistent with a proposed reciprocal regulatory relationship among EGFRvIII, *RP11-30P6.6*, and the tested miRNAs.

### DNA methylation contributes partly to the regulation of *RP11-30P6.6*–associated miRNAs

Considering the methylating activity of NNK and arecoline, we further determined whether methylation plays a role in the regulation of *RP11-30P6.6*, miRNAs, and *EGFRvIII* expression. We then treated the cells with a DNA demethylating agent, 5-aza-dC. The results of qRT-PCR analysis revealed the expression of miR-197-3p, miR-3941, miR-11181-3p, and miR765 following treatment, indicating that promoter methylation contributes partly to their silencing (Figure S11). In addition, no significant difference in *RP11-30P6.6* expression was noted following 5-aza-dc treatment. Consistently, EGFRvIII expression decreased following 5-aza-dC treatment in DOK-NNKL and DOK-AL cells, whereas RP11-30P6.6 expression was not significantly altered. These findings are consistent with a contribution of DNA methylation to the regulation of the miRNA–EGFRvIII axis independently of detectable changes in RP11-30P6.6 under these conditions.

## Discussion

OSCC is a major public health burden in South and Southeast Asia, particularly in Taiwan, where its incidence and mortality remain high [1, 50]. Areca (betel) nut chewing and cigarette smoking are predominant risk factors that act synergistically to drive oral carcinogenesis and poor outcomes [22, 38]. In endemic regions, OSCC frequently presents at advanced stages, highlighting delayed detection and the need for improved prevention and therapeutic strategies [29, 31, 49].

EGFR is a central oncogenic driver in OSCC and other epithelial malignancies, which regulates proliferation, survival, and migration through the RAS–RAF–MEK–ERK, PI3K–AKT, and JAK–STAT cascades [63]. Dysregulated EGFR signaling is a hallmark of OSCC and GBM, wherein EGFR amplification and structural alterations, including the constitutively active EGFRvIII variant, drive tumor progression, treatment resistance, and poor prognosis [10, 34, 45, 52, 58]. This is one of the reasons why clinical responses to EGFR-targeted therapies in OSCC and GBM remain modest, indicating that additional regulatory layers, particularly isoform diversity and RNA-based control, may influence EGFR dependency.

In this study, we identified a carcinogen-associated splicing–ceRNA regulatory axis that governs *EGFRvIII* expression in OSCC. We show that tobacco- and areca-derived carcinogens, NNK and arecoline, reprogram RNA-processing and ncRNA networks, thereby biasing EGFR signaling toward EGFRvIII. These findings provide a mechanistic framework linking environmental exposure to isoform-specific oncogenic output and may establish an “EGFRvIII-permissive” state in carcinogen-exposed epithelial cells. At the genomic and epigenomic levels, NNK and arecoline may act as the drivers of cellular reprogramming. NNK induces DNA adduct formation and activates DNA damage responses [15, 16, 28, 36], whereas arecoline triggers DNA damage–associated stress and disrupts the p53-dependent repair pathways [51]. These effects are associated with epigenetic alterations, including changes in DNA methylation and dysregulation of ncRNA networks in head and neck cancers [2, 30, 55]. Accordingly, the induction of EGFRvIII observed in our models reflects a cumulative “genome–epigenome–post-transcriptional” remodeling rather than a transient transcriptional response. Carcinogen exposure was associated with remodeling of the RNA-processing landscape, including altered expression of several regulators such as SRSF1, SRSF7, PTBP1, DDX21, and DDX60, together with increased EGFRvIII expression and exon 1–exon 8 junction-containing transcripts. Among these factors, SRSF1 showed a functional association with EGFRvIII regulation, as its depletion reduced EGFRvIII expression.

Given that multiple identified splicing factors, including SRSF1, DDX60, and PTBP1, are core components of the general splicing machinery, our observations are consistent with a model in which *RP11-30P6.6* may contribute to an RNA-processing environment involving splicing regulators and *EGFRvIII*-associated RNA processing.

Although chronic carcinogen exposure is expected to induce broader transcriptomic remodeling and alterations in RNA-processing pathways, we could not distinguish the relative contribution of these global changes from *EGFRvIII*-specific regulatory mechanisms. Nevertheless, the observation that *EGFRvIII* induction also occurred following short-term (72–96 h) NNK or arecoline exposure suggests that the observed *EGFRvIII*-associated changes are unlikely to merely represent a secondary consequence of long-term carcinogen adaptation. Future studies will be required to further dissect the relative contribution of *EGFRvIII*-specific regulation and broader transcriptomic remodeling during carcinogen-induced transformation. Given that multiple identified splicing factors are core components of the general splicing machinery, our findings are consistent with a model in which *RP11-30P6.6* participates in *EGFRvIII*-associated RNA processing and contributes to the regulation of alternative splicing.

These findings are consistent with a model in which carcinogen exposure-induced changes in splicing factor activity lower the barrier for coordinated multi-exon skipping, which is consistent with the growing evidence that aberrant alternative splicing is a disease-defining and therapeutically tractable feature of OSCC [8, 17, 25, 32, 35, 47]. Our data support a model in which *RP11-30P6.6* functions upstream of SRSF1 to facilitate *EGFRvIII*-associated RNA processing. Nevertheless, this study does not distinguish whether *RP11-30P6.6* directly regulates *EGFR* splice-site selection by promoting the recruitment of SRSF1 to EGFR pre-mRNA or indirectly enhances *EGFRvIII* expression via modulation of SRSF1 abundance or broader RNA-processing mechanisms. In addition, the functional domains of *RP11-30P6.6* responsible for these regulatory effects remain undefined. Future studies examining SRSF1 occupancy on *EGFR* pre-mRNA together with domain-mapping analyses of *RP11-30P6.6* will help clarify the molecular mechanism underlying *RP11-30P6.6*-mediated regulation of EGFR alternative splicing.

Although EGFRvIII is frequently associated with genomic deletions or structural rearrangements, our data do not fully support a purely DNA-driven origin. Instead, we propose an integrative “gain-control” model, in which genomic permissiveness provides the substrate for EGFRvIII, whereas carcinogen exposure-induced remodeling of the splicing machinery amplifies transcript maturation and steady-state abundance. Our findings do not exclude the contribution of genomic rearrangements but suggest that RNA-processing mechanisms can modulate the abundance of *EGFRvIII*-compatible transcripts once a permissive genomic context exists. This framework helps explain the variability in EGFRvIII detection across HNSCC cohorts and identifies carcinogen exposure history as a key modifier of the EGFRvIII output [58]. A second layer of regulation is provided by the lncRNA *RP11-30P6.6*, which functions as a carcinogen-responsive factor linking splicing to posttranscriptional regulation of *EGFRvIII*. *RP11-30P6.6* is associated with AGO2- and splicing-related fractions and may modulate candidate miRNAs that showed differential responsiveness of *EGFRvIII* relative to WT-*EGFR* under the conditions tested, including miR-197-3p, miR-10392-3p, and miR-765. The reporter assays showed greater responsiveness of the EGFRvIII CDS construct to these miRNAs than of the WT-EGFR CDS construct, while AGO2 immunoprecipitation supported the association of these miRNAs with *RP11-30P6.6*-containing complexes. Reciprocal regulation between *EGFRvIII* and *RP11-30P6.6* further supports a ceRNA-like role for *RP11-30P6.6* in EGFRvIII regulation. These findings are consistent with a coupled splicing–ceRNA model in which carcinogen exposure-driven splicing generates *EGFRvIII* transcripts, whereas *RP11-30P6.6* attenuates miRNA-mediated repression to stabilize EGFRvIII protein expression. This dual-layer architecture may confer regulatory robustness, such that perturbation of a single regulatory layer may not be sufficient to fully suppress EGFRvIII output. The differential responsiveness of the WT-EGFR and EGFRvIII CDS reporters also highlights the context-dependent nature of miRNA-mediated regulation. Although the downstream nucleotide sequences corresponding to the predicted miRNA-responsive regions are retained in both WT-EGFR and EGFRvIII, the physical presence of a potential target sequence does not necessarily imply equivalent functional repression. miRNA-mediated targeting is influenced not only by sequence complementarity but also by target-site accessibility, local RNA secondary structure, the surrounding nucleotide context, and the local ribonucleoprotein environment. Experimental studies have shown that reduced target-site accessibility can markedly diminish miRNA-mediated repression, whereas flanking nucleotide context can substantially alter Argonaute–miRNA target affinity; nearby RNA-binding proteins can further modulate miRNA-targeting efficacy through effects on RNA accessibility and AGO occupancy [14, 23, 24, 37]. Because deletion of exons 2–7 changes the overall sequence context of the EGFRvIII transcript while retaining the shared downstream sequences, such context-dependent effects may contribute to the greater responsiveness of the EGFRvIII reporter observed in our study. However, RNA secondary structure, target-site accessibility, RBP occupancy, and site-specific AGO occupancy were not directly examined for WT-EGFR versus EGFRvIII, and the molecular basis of this differential responsiveness therefore remains to be determined.

To investigate additional upstream regulatory inputs into this axis, we treated the cells with the DNA demethylating agent 5-aza-dC. Reactivation of miR-197-3p, miR-3941, and miR-11181-3p following treatment was consistent with epigenetic suppression of these miRNAs. Simultaneously, EGFRvIII levels were reduced, whereas core splicing factors remained largely unaffected. This dissociation suggests that DNA methylation may not primarily regulate the spliceosomal abundance but influences EGFRvIII expression through the modulation of miRNA-mediated repression. These findings extend our model by incorporating an epigenetic layer in which DNA methylation may influence the availability of the tested miRNAs, thereby contributing to the regulation of EGFRvIII expression. This is consistent with the finding that exposure to carcinogens, including NNK, modulates miRNA expression profiles in squamous cancer cells [8].

A limitation of this study was the absence of conventional in vivo genetic models, reflecting the human-specific nature of *EGFRvIII* and *RP11-30P6.6*, which lack clear murine orthologs. We, therefore, focused on in-depth characterization of this carcinogen-responsive regulatory circuit in human cellular systems, complemented by analyses of patient-derived datasets. By analyzing TCGA-HNSC datasets, we found upregulation of RNA splicing–related genes, particularly members of the SRSF family (Figure S12), in primary tumors, which highlights the clinical relevance of this regulatory axis. Moreover, despite being an attractive tumor-specific target, EGFRvIII-directed therapies have shown limited and inconsistent clinical benefits. Multiple approaches, including the use of tyrosine kinase inhibitors, monoclonal antibodies, peptide vaccines, and adoptive cell therapies, have been explored; however, their efficacy has been constrained by intratumoral heterogeneity, antigen loss, and adaptive resistance. Notably, the phase III ACT IV trial demonstrated that the EGFRvIII-targeting peptide vaccine rindopepimut failed to improve overall survival in patients with newly diagnosed GBM, underscoring the difficulty of targeting EGFRvIII alone as a stable therapeutic antigen [57]. EGFRvIII-directed CAR-T cell therapy can mediate tumor infiltration but is frequently accompanied by decreased EGFRvIII expression and induction of immunosuppressive pathways, suggesting dynamic antigen modulation and adaptive tumor escape [39]. Together, these findings indicate that targeting the EGFRvIII protein alone makes it difficult to achieve durable therapeutic responses. These limitations highlight the importance of investigating the upstream regulatory mechanisms that sustain EGFRvIII expression. In this context, we identified an *RP11-30P6.6*–miRNA–SRSF1 axis that coordinates RNA-processing events linked to EGFRvIII expression. These results suggest that splicing-associated mechanisms may contribute to the maintenance of EGFRvIII, particularly under carcinogen exposure, thereby providing a regulatory layer that is not tackled by conventional receptor-targeted therapies. Therefore, targeting this upstream RNA regulatory circuitry may provide a complementary strategy to reduce EGFRvIII expression and improve treatment outcomes. This warrants further research. Another limitation of this study concerns the induction of widespread transcriptomic and RNA-processing alterations upon chronic carcinogen exposure. Therefore, although our data demonstrate a clear association between chronic carcinogen exposure and *EGFRvIII*-associated transcript remodeling, the contribution of broader RNA-processing changes cannot be completely distinguished from EGFRvIII-specific regulation. In addition, this study does not distinguish whether *RP11-30P6.6* directly regulates EGFR splice-site selection or indirectly influences EGFRvIII expression through the modulation of SRSF1 abundance or broader RNA-processing mechanisms. Future studies examining SRSF1 occupancy on *EGFR* pre-mRNA and defining the functional domains of *RP11-30P6.6* are required to clarify the underlying molecular mechanism.

## Conclusion

This study unraveled a carcinogen exposure–driven RNA regulatory framework that regulates *EGFRvIII* expression in OSCC and GBM cells. We show that tobacco- and areca-derived carcinogens coordinate splicing and ncRNA networks to promote *EGFRvIII* expression, establishing a regulatory state in which oncogenic isoform expression is actively maintained rather than solely dictated by genomic alteration.

## Methods

- Protein extraction and western blot analysis

- CCK-8 cell proliferation assay

- Colony formation assay

- Transwell migration assay

- Reverse transcription and quantitative PCR

- MS2 binding-site cassette and EGFR minigene construction

- Plasmid construction: NLS–FLAG–MCP–GST fusion protein

## Supplementary Information

Supplementary Material 1. Fig. S1 NNK and arecoline induce EGFRvIII and splicing regulators in glioblastoma cells. S2 Schematic overview of canonical EGFRvIII, the PacBio Iso-Seq transcript PB.11430.142, and the lncRNA RP11-30P6.6. Fig. S3 RP11-30P6.6 promotes the proliferation, colony formation and migration of OSCC cells. Fig. S4 RP11-30P6.6 regulates EGFRvIII expression in DOK-AL and DOK-NNKL cells. Fig. S5 Identification of splicing factors regulating *EGFRvIII *exon skipping. Fig. S6 SRSF1 promotes malignant phenotypes in DOK-derived cells. Fig. S7 Schematic representation of putative miRNA-binding sites mapped within the EGFRvIII coding sequence. Fig.8 Co-transfection of *RP11-30P6.6 *partially attenuates candidate miRNA-mediated suppression of *EGFRvIII* expression. Fig.9 *RP11-30P6.6* modulates the expression of candidate miRNAs associated with EGFRvIII regulation. Fig. 10 EGFRvIII suppresses the expression of regulatory miRNAs. Fig. 11 DNA demethylation alters the expression of selected miRNAs and EGFRvIII. Fig. 12 Expression of splicing-related genes in normal and primary tumor tissues (TCGA-HNSC)

Supplementary Material 2. Table S1 Primers used for qRT-PCR

## Data Availability

The RNA sequencing data generated in this study were deposited in the Gene Expression Omnibus (GEO) database under accession number GSE325659. The data are currently under controlled access during peer review and are available to reviewers via a secure token. All processed data supporting the findings of this study, including normalized expression matrices and raw count tables, are included in the GEO submission. The data will be made publicly available upon publication. All other data supporting the findings of this study are available within the paper and its Supplementary files or from the corresponding author upon reasonable request.

## Abbreviations

AGO2: Argonaute 2
AL: Arecoline-tolerant
BSA: Bovine serum albumin
CCS: Circular consensus sequencing
ceRNA: Competing endogenous RNA
DOK: Dysplastic oral keratinocyte
EGFR: Epidermal growth factor receptor
EGFRvIII: Epidermal growth factor receptor variant III
FSM: Full-splice match
GBM: Glioblastoma multiforme
ISM: Incomplete-splice match
JCRB: Japanese Collection of Research Bioresources
LncRNA: Long noncoding RNA
miRNA: MicroRNA
ncRNA: Noncoding RNA
NIC: Novel in catalog
NNC: Novel not in catalog
NNK: Nicotine-derived nitrosamine ketone
NNKL: NNK-tolerant
OSCC: Oral squamous cell carcinoma
RISC: RNA-induced silencing complex
RNA-FISH: RNA fluorescence in situ hybridization
RNP: Ribonucleoprotein
