# Supplementary material for: RNA splicing factors and long noncoding RNA reciprocally regulate EGFRvIII expression in human cancer cells
Source: J Biomed Sci. 2026 Nov 11;33:95. doi: 10.1186/s12929-026-01298-z (PMC13753392; doi:10.1186/s12929-026-01298-z)
Supplement: Supplementary file 1 [file 12929_2026_1298_MOESM1_ESM.docx]

**Supplementary Materials for**

**RNA splicing factors and long noncoding RNA reciprocally regulate EGFRvIII expression in human cancer cells**

**Authors:**

Hsuan-Yu Peng, Jang-Yang Chang*

TMU Research Center of Cancer Translational Medicine, Taipei Medical University Hospital, College of Medicine, Taipei Medical University, Taipei, 110301 Taiwan (R.O.C.)

***Correspondence: Professor Jang-Yang Chang, MD**

**E‐mail: jychang@tmu.edu.tw; jychang@nhri.edu.tw**

**TMU Research Center of Cancer Translational Medicine, Taipei Medical University Hospital, College of Medicine, Taipei Medical University, No. 252, Wuxing St., Xinyi Dist., Taipei, 110301 Taiwan (R.O.C.)**

**This file includes:**

**Methods**

**Supplementary Figures 1 to 12**

**Methods**

**Protein extraction and western blot analysis**

Cells were lysed in RIPA buffer [50 mM Tris-HCl (pH 7.4), 150 mM NaCl, 1% NP-40, 0.1% SDS, 1 mM PMSF, 1 mM Na₃VO₄] supplemented with protease and phosphatase inhibitor cocktail (1:1,000; Sigma-Aldrich, St Louis, MO, USA). Protein concentrations were measured using the BCA Protein Assay Kit (Thermo Scientific, USA). Equal amounts of protein (10–30 μg per lane) were resolved by SDS–PAGE on 10% polyacrylamide gels and transferred onto polyvinylidene fluoride (PVDF) membranes (Pall Life Sciences, Glen Cove, NY, USA). Membranes were blocked and incubated overnight at 4 °C with primary antibodies against EGFR (#4267, Cell Signaling Technology, USA), EGFRvIII (31-1305-00, RevMAb Biosciences, USA), SRSF1 (A4091, ABclonal), SRSF7 (#82637, Cell Signaling Technology), DDX21 (#75762, Cell Signaling Technology), DDX60 (GTX32082, GeneTex, Irvine, CA, USA), PTBP1 (#57246, Cell Signaling Technology), and GAPDH (GTX100118, GeneTex). GAPDH served as a loading control. After washing, membranes were incubated with HRP-conjugated secondary antibodies for 1 h at room temperature. Protein bands were visualized using enhanced chemilluminescence (ECL; PerkinElmer, Waltham, MA, USA) and imaged with an e-BLOT WB/SDS-PAGE Touch Imager (eBLOT, Shanghai, China). Band intensities were quantified using ImageJ software (NIH, Bethesda, MD, USA), and target protein levels were normalized to GAPDH.

**CCK-8 cell proliferation assay**

OSCC cells were transduced with RP11-30P6.6, SRSF1, or empty control vectors and cultured for 72 h. Stable cell lines were generated by drug selection for a further 7 days. After selection, equal numbers of cells were seeded into 96-well plates at identical densities and incubated for up to 72 h. For knockdown experiments, OSCC cells were transfected with siRNA targeting RP11-30P6.6 (si30P6.6), SRSF1(siSRSF1), or a non-targeting control (NC) and cultured for 72 h before seeding equal numbers of cells into 96-well plates for an additional 72 h. Cell proliferation was assessed using the Cell Counting Kit-8 (CCK-8; Sigma-Aldrich, St Louis, MO, USA) according to the manufacturer’s instructions. Briefly, 10 µL CCK-8 reagent was added to each well, and plates were incubated at 37 °C for 2 h. Absorbance at 450 nm was measured using a SpectraMax 250 microplate reader (Molecular Devices, CA, USA).

**Colony formation assay**

For clonogenic assays, DOK, DOK AL, DOK NNKL, Cal-27, HSC-2, and HSC-3 cells (150 cells per well) and SCC-9 cells (300 cells per well) were seeded into 6-well plates and cultured for 7 to 10 days at 37 °C in a humidified incubator with 5% CO₂. Colonies were fixed with 100% ethanol, stained with 1% crystal violet, and counted under a light microscope.

**Transwell migration assay**

Cell migration was evaluated using 24-well Transwell inserts (PET membrane, 8-µm pores; SPL Life Sciences, Cat# 36224, Korea) without extracellular matrix coating. OSCC cells were serum-starved for 6–12 h prior to seeding. For migration assays, 5 × 10⁴ cells in 200 µL serum-free medium were added to the upper chamber, and 600 µL medium containing 2% FBS was added to the lower chamber as chemoattractant. Plates were incubated at 37 °C, 5% CO₂ for 24 h, ensuring that cells did not reach confluence on the lower membrane surface.

Non-migrated cells on the upper membrane surface were removed with a cotton swab. Membranes were washed with PBS, fixed in 100% ethanol at 4 °C for 60 min, and stained with 0.1% crystal violet in 20% methanol for 30 min. Excess stain was removed by PBS rinses. Cells on the lower membrane surface were visualized at 200× magnification on an inverted microscope (Olympus, Tokyo, Japan), and images were analyzed using Imaging Research software (Ontario, Canada) to quantify migrated cells.

**Reverse transcription and quantitative PCR**

For miRNA profiling, 5 μg of total RNA was polyadenylated using E. coli poly(A) polymerase (KACTUS Bio, Cat# PLA-EE101) in the presence of 25 mM MnCl₂ and ATP at 37 °C for 30 min. The poly(A)-tailed RNA was then annealed with an oligo(dT) adaptor primer (5′-CAG TGC AGG GTC CGA GGT CAG AGC CAC CTG GGC AAT TTT TTT TTT TVN NNN N-3′) at 60 °C for 5 min and reverse-transcribed using the RevertAid Reverse Transcription Kit (Thermo Fisher, Cat# K1691) at 42 °C for 60 min. A universal reverse primer (5′-CAG TGC AGG GTC CGA GGT-3′) was used for subsequent qPCR detection of miRNAs. Quantitative PCR (qPCR) was performed using ChamQ Universal SYBR qPCR Master Mix (Q711-02, Vazyme, Nanjing, China) on a QuantStudio 3 Real-Time PCR System (Applied Biosystems, Life Technologies, Carlsbad, CA, USA). GAPDH was used as the internal control for mRNA expression, and U6 small nuclear RNA (snRNA) was used as the internal control for miRNA expression. Relative expression levels were calculated using the 2^−ΔΔCt method following normalization to the respective internal controls. **Primer sequences are listed in Supplementary Table S1**.

**MS2 binding-site cassette and EGFR minigene construction**

To enable MS2-based RNA affinity purification, MS2-tagged EGFR minigene plasmids were generated in the pcDNA3.1 (+) backbone (Thermo Fisher Scientific). An in-frame stop codon (TAA) was inserted immediately downstream of the EGFR coding region, followed by a synthetic MS2 binding-site cassette containing six tandem MS2 hairpins (MS2×6). Each hairpin contains the canonical MS2 translational operator (TR) motif (DNA: 5′-ACATTGAGGATTACCCATGT-3′; RNA: 5′-ACAUUGAGGAUUACCCAUGU-3′), which is specifically recognized by the MS2 coat protein (MCP). Individual hairpins were separated by short neutral spacer sequences to minimize unfavorable secondary structures and maintain efficient MCP binding. The MS2×6 cassette was flanked by outward-facing BsaI recognition sites (GGTCTC) to allow modular replacement of the RNA-tagging cassette.

Two EGFR minigene constructs were generated.

The first construct, EGFR_minigene_Ex1–Inx1–…–Ex7–Inx7–Ex8_MS2x6_stop_BsaI (~5.4 kb insert), contains EGFR exons 1–8 with truncated intronic regions (~300 bp flanking each exon) to preserve key splice-regulatory elements. The second construct, EGFR_minigene_Ex1–Inx1–Ex2–Ex7–Inx7–Ex8_MS2x6_stop_BsaI (~2.5 kb insert), contains exons 1, 2, 7 and 8 with intronic sequences adjacent to exons 2 and 7, modelling the EGFRvIII-type exon 1–exon 8 splicing junction. All inserts were directionally cloned between the NheI and XhoI sites of pcDNA3.1 (+), and the integrity of all junctions and complete inserts was confirmed by Sanger sequencing.

**Plasmid construction: NLS–FLAG–MCP–GST fusion protein**

For GST-based MS2-TRAP assays, a nuclear-localized MS2 coat protein fusion plasmid (pcDNA3.1_NLS–FLAG–MCP–GGGGS–GST) was constructed in the pcDNA3.1 (+) backbone. The expression cassette contains a Kozak sequence (GCCACC) followed by a start codon, an N-terminal SV40 nuclear localization signal (NLS), a FLAG epitope (DYKDDDDK), the bacteriophage MS2 coat protein (MCP), a flexible glycine–serine linker (GGGGS), and a C-terminal glutathione S-transferase (GST) domain. The coding sequence was synthesized as a single open reading frame and codon-optimized for mammalian expression. The insert was cloned in-frame between the NheI and XhoI sites of pcDNA3.1 (+), generating the plasmid pcDNA3.1_NLS–FLAG–MCP–GGGGS–GST (also referred to as pcDNA3.1(+)-NLS-GST-MCP). The 5′ region of the insert begins with

5′-GCTAGCGCCACCATGCCGAAGAAGAAGCGCAAGGTGGACTACAAGGACGACGACGACAAG-3′ which encodes the Kozak sequence, NLS, and FLAG tag.

**Supplementary Data**

**Supplementary Data Figure 1 | NNK and arecoline induce EGFRvIII and splicing regulators in glioblastoma cells.**

(A) Immunoblot analysis of EGFRvIII and EGFR in U87 and A172 cells treated with 10 μM NNK or 50 μM arecoline for 96 h.

(B) qRT–PCR analysis of EGFRvIII mRNA expression in U87 cells treated with 10 μM NNK or 50 μM arecoline for 96 h.

**Supplementary Data Figure 2 |** **Schematic overview of canonical EGFRvIII, the PacBio Iso-Seq transcript PB.11430.142, and the lncRNA RP11-30P6.6.**

**(A)** Schematic representation of wild-type EGFR and canonical EGFRvIII transcript structures. Wild-type EGFR contains exons 1–8, whereas canonical EGFRvIII contains the characteristic exon 1–exon 8 junction, with exons 2–7 absent from the mature transcript. A simplified representation of the exon 1–exon 8 junction and representative Sanger sequencing results confirming the junction sequence are shown.

**(B)** Schematic representation of the PacBio Iso-Seq transcript PB.11430.142 identified in this study and its predicted protein product. The predicted open reading frame of PB.11430.142 initiates at MEEDGVR. Its predicted protein sequence was compared with those of wild-type EGFR and canonical EGFRvIII to assess sequence similarity. PB.11430.142 is presented as an Iso-Seq-derived transcript identified in this study and is not designated as the canonical EGFRvIII transcript.

**(C)** Transcript structure of the lncRNA RP11-30P6.6 (ENST00000455071) based on the Ensembl GRCh37 annotation (ENSG00000234155). RP11-30P6.6 is transcribed from the reverse strand and consists of two exons separated by one intron. Exon and intron lengths, the spliced transcript length (2,513 bp), and the genomic transcript span (2,968 bp) are indicated. Exon and intron lengths are shown schematically and are not drawn to scale.

**(D)** Representative amino acid sequence alignment of wild-type EGFR, canonical EGFRvIII, and PB.11430.142. The alignment illustrates the sequence relationships among the three predicted protein products, including the predicted translation initiation region of PB.11430.142.

**Supplementary Data Figure 3 | RP11-30P6.6 promotes the proliferation, colony formation and migration of OSCC cells.**

(A) Growth curves of Cal-27 and SCC-9 cells stably overexpressing RP11-30P6.6 or empty vector, measured by CCK-8 at 24, 48, and 72 h.

(B) Growth curves of HSC-2 and HSC-3 cells transfected with siRP11-30P6.6 or non-targeting control (NC), assessed by CCK-8 at 24, 48, and 72 h.

(C) Colony formation assays of Cal-27 and SCC-9 cells overexpressing RP11-30P6.6 or vector (7 days).

(D) Colony formation assays of HSC-2 and HSC-3 cells transfected with siRP11-30P6.6 or NC (7 days).

(E–F) Transwell migration assays (24 h) of Cal-27 and SCC-9 cells (E) or HSC-2 and HSC-3 cells (F) under the indicated conditions. Scale bars, 20 μm.

(G) Growth curves of DOK, DOK-AL, and DOK-NNKL cells transfected with siRP11-30P6.6 or NC, measured by CCK-8 at 24, 48, and 72 h.

(H) Colony formation assays of the indicated cells transfected with siRP11-30P6.6 or NC (7 days).

(I) Transwell migration assays (24 h) of the indicated cells. Scale bars, 20 μm.

Data are presented as mean ± SD from three independent experiments. Statistical significance was determined using unpaired two-tailed Student’s t-test (two groups) or one-way ANOVA with appropriate post hoc tests (≥3 groups); *P < 0.05, **P < 0.01, ***P < 0.001.

**Supplementary Data Figure 4 | RP11-30P6.6 regulates EGFRvIII expression in DOK-AL and DOK-NNKL cells.**

(A) qRT–PCR analysis of RP11-30P6.6 expression in DOK, DOK-AL, and DOK-NNKL cells transfected with siRP11-30P6.6 or negative control (NC) for 72 h.

(B) Immunoblot analysis of EGFR and EGFRvIII in the same samples. GAPDH was used as a loading control. Densitometric quantification normalized to GAPDH is shown beneath the blots.

**Supplementary Data Figure 5 | Identification of splicing factors regulating EGFRvIII exon skipping.**

(A) RT–PCR and qRT–PCR analysis of EGFR minigene transcripts in HEK293T cells transfected with siRNAs targeting PTBP1, SRSF1, SRSF7, DDX21, or DDX60 for 48 h.

(B) Immunoblot analysis of the indicated proteins in DOK-AL, DOK-NNKL, and HSC-3 cells following siRNA treatment for 72 h.

(C) Immunoblot analysis of PTBP1, SRSF1, SRSF7, DDX21, and DDX60 in DOK-AL and DOK-NNKL cells transfected with siRP11-30P6.6 or negative control (NC) for 72 h. GAPDH was used as a loading control.

**Supplementary Data Figure 6 | SRSF1 promotes malignant phenotypes in DOK-derived cells.**

(A) Growth curves of DOK, DOK-AL, and DOK-NNKL cells following transfection with siSRSF1 or negative control (NC).

(B) Colony formation assays of the indicated cells transfected with siSRSF1 or NC (7 days).

(C) Transwell migration assays (24 h) of the indicated cells. Scale bars, 20 μm.

Data are presented as mean ± SD from three independent experiments.

**Supplementary Data Figure 7 |** **Schematic representation of putative miRNA-binding sites mapped within the EGFRvIII coding sequence.**

(A) Linear map of the 3,093-nt EGFRvIII coding sequence. Exon 1 spans nt 1–167, whereas exon 8 spans nt 168–293, generating the characteristic exon 1–exon 8 junction. The predicted binding sites of hsa-miR-197-3p, hsa-miR-3941, hsa-miR-11181-3p, hsa-miR-10392-3p, and hsa-miR-765 are indicated at their respective nucleotide positions along the EGFRvIII sequence. All positions are plotted using the same linear 1–3093 nt coordinate scale. (B) Summary of the mature miRNA sequences and their predicted seed locations within the EGFRvIII coding sequence. (C) Seed-centered duplex alignments between the indicated miRNAs and their corresponding EGFRvIII target sites. miRNA seed sequences are shown in the 3′→5′ orientation, whereas the complementary EGFRvIII target sequences are displayed in the 5′→3′ orientation. Vertical bars indicate canonical Watson–Crick base pairing, a colon indicates a G–U wobble pair, and blank spaces indicate mismatches. These predicted sites are mapped to the EGFRvIII coding sequence for illustration and are not considered EGFRvIII-specific nucleotide sequences, as the corresponding downstream sequences are also present in WT-EGFR.

**Supplementary Data Figure 8 | Co-transfection of RP11-30P6.6 partially attenuates candidate miRNA-mediated suppression of EGFRvIII expression.**

(A) Immunoblot analysis of EGFRvIII, EGFR, and GAPDH in HSC-3 cells co-transfected with either an RP11-30P6.6 expression plasmid or the corresponding vector control together with a negative-control mimic or the indicated candidate miRNA mimics (miR-197-3p, miR-3941, miR-10392-3p, miR-11181-3p, or miR-765; 50 nM) for 72 h. GAPDH served as the loading control.

(B) qRT-PCR analysis confirming successful overexpression of the indicated miRNAs following co-transfection. RP11-30P6.6 expression was also measured to confirm maintenance of RP11-30P6.6 overexpression throughout the experiment. Data are presented as the mean ± SD from independent experiments.

**Supplementary Data Figure 9 | RP11-30P6.6 modulates the expression of candidate miRNAs associated with EGFRvIII regulation.**

(A) qRT–PCR analysis of RP11-30P6.6 and candidate miRNAs (miR-197-3p, miR-11181-3p, miR-765, miR-10392-3p, and miR-3941) in HSC-3 cells following transfection with siRP11-30P6.6 for 72 h.

(B) qRT–PCR analysis of the same RNAs in SCC-9 cells overexpressing RP11-30P6.6. Expression levels were normalized to GAPDH (RP11-30P6.6) or U6 (miRNAs).

**Supplementary Data Figure 10 | EGFRvIII suppresses the expression of regulatory miRNAs.**

(A) Immunoblot analysis of EGFR and EGFRvIII in Cal-27 cells transfected with EGFRvIII or vector control. GAPDH serves as a loading control.

(B–C) qRT–PCR analysis of RP11-30P6.6 and the indicated candidate miRNAs under the indicated conditions. Expression was normalized to U6 (miRNAs) or GAPDH (lncRNA).

(D) qRT–PCR analysis of RP11-30P6.6 and the indicated miRNAs. Expression was normalized to U6.

**Supplementary Data Figure 11 | DNA demethylation alters the expression of selected miRNAs and EGFRvIII.**

(A) qRT–PCR analysis of miRNA expression in cells treated with 5-aza (5 μM) for 7 days. Expression levels were normalized to U6.

(B) Immunoblot analysis of EGFR and EGFRvIII in DOK, DOK-AL, and DOK-NNKL cells treated with 5-aza (5 μM) for 7 days (vehicle control). GAPDH was used as a loading control. Densitometric quantification normalized to GAPDH is presented relative to vehicle.

**Supplementary Data Figure 12 | Expression of splicing-related genes in normal and primary tumor tissues (TCGA-HNSC)**

Box-and-whisker plots generated from the UALCAN portal (TCGA-HNSC cohort) showing mRNA expression levels of the indicated splicing-related genes in normal versus primary tumor tissues. Data are derived from The Cancer Genome Atlas (TCGA) head and neck squamous cell carcinoma (HNSC) dataset.
