# Supplementary material for: RNA splicing factors and long noncoding RNA reciprocally regulate EGFRvIII expression in human cancer cells
Source: J Biomed Sci. 2026 Nov 11;33:95. doi: 10.1186/s12929-026-01298-z (PMC13753392; doi:10.1186/s12929-026-01298-z)
Supplement: Supplementary file 2 [file 12929_2026_1298_MOESM2_ESM.docx]

**Supplementary Data Table**

**Supplementary Data Table 1. Primers used for qRT–PCR**

| **Name** | **Primer sequence (5′→3′)** |
| --- | --- |
| EGFRvIII real-time PCR Forward | CTC TGG AGG AAA AGA AAG GTA A |
| EGFRvIII real-time PCR Reverse | AGG CCC TTC GCA CTT CTT AC |
| RP11-30P6 (2) Forward | CAC AGC TCT GTA GTG GCA CA |
| RP11-30P6 (2) Reverse | CGC CTT TCT GGT GAC TCC AA |
| RP11-30P6 (3) Forward | TGC TGC TTA AGT CTT GCC CA |
| RP11-30P6 (3) Reverse | GGC TTC GGG GGT TAT TTC CT |
| DDX60 Forward | CAG CTC CAA TGA AAT GGT GCC |
| DDX60 Reverse | CTC AGG GGT TTA TGA GAA TGC C |
| DDX21 Forward | TCA TCA AGG ACG CAC TAT CAT CT |
| DDX21 Reverse | CCT TTC AGG GTG ATT TCC CTT T |
| DDX58 Forward | TGT GCT CCT ACA GGT TGT GGA |
| DDX58 Reverse | CAC TGG GAT CTG ATT CGC AAA A |
| RASSF3 Forward | GCC TGC AAG CTC TCA GAC C |
| RASSF3 Reverse | TAG GCT GTG TAG CGC CTC TT |
| SNRNP25 Forward | CTG CGA TCT GCC GAT CCA G |
| SNRNP25 Reverse | CAC TCG GAC CGT CAT TGC C |
| SNRNP40 Forward | TGT GGA ATG TCT ATG GTG ACT GT |
| SNRNP40 Reverse | GAG AAA AGC ATA CTG CCA TCT GT |
| SRSF7 Forward | GGA AAG GTG ATT TGT GGC TCC |
| SRSF7 Reverse | GGT CTA TCA AAA CGT GAT CTC CG |
| PRPF4 Forward | GTT GTC GGT ACT GAT GCC TTG |
| PRPF4 Reverse | GCA ATC CAT AGT CTT GCC ACC |
| DDX10 Forward | GTG CGG AGC TTC AAT CGC T |
| DDX10 Reverse | CCT GCC ATT CGG GTT TCT TCA |
| SF3B3 Forward | GCC ATT CAT GGA AAC TTT TCT GG |
| SF3B3 Reverse | GTG AGT AGG GTA TGT ACT TTG CC |
| IGSF3 Forward | AGG GCT CCC ACA TCA CTA TCT |
| IGSF3 Reverse | GAA GGC AGG TAA ATG GAC CAC |
| PTBP1 Forward | CTC CAA GTT CGG CAC AGT GTT G |
| PTBP1 Reverse | CAG GCG TTG TAG ATG TTC TGC C |
| SRSF1 Forward | CCG CAG GGA ACA ACG ATT G |
| SRSF1 Reverse | GCC GTA TTT GTA GAA CAC GTC CT |
| GAPDH Forward | GTC TCC TCT GAC TTC AAC AGC G |
| GAPDH Reverse | ACC ACC CTG TTG CTG TAG CCA A |
| hsa-miR-197-3p Forward | TTA TGG GGC TGG GGC CGG |
| hsa-miR-3941 Forward | GCG CTT CAC CAC CTT CT |
| hsa-miR-11181-3p Forward | GCG CTT ACA CAC AAC TGA |
| hsa-miR-10392-3p Forward | GCG CAG GAG GAG GAG |
| hsa-miR-765 Forward | CCG GCC CCG CC |
| bdi-miR159-5p Forward | GCG GCG AGC TCC CTT CGA T |
| U6 Forward | CGC AAG GAT GAC ACG CAA ATT C |
| Universal reverse primer (polyA) | CAG TGC AGG GTC CGA GGT |
